# Supplementary material for: Chagas Disease Megaesophagus Patients Carrying Variant MRPS18B P260A Display Nitro-Oxidative Stress and Mitochondrial Dysfunction in Response to IFN-γ Stimulus
Source: Biomedicines. 2022 Sep 7;10(9):2215. doi: 10.3390/biomedicines10092215 (PMC9496350; doi:10.3390/biomedicines10092215)
Supplement: Supplementary file 1 [file biomedicines-10-02215-s001.zip › Supplemental Table S1.pdf]

Supplemental Table S1: List of the genes contained in the Mitochondrion Gene Ontology term and Mitocarta 2.0.

Gene	EnsemblID	Name
AADAT	ENSG00000109576	aminoadipate aminotransferase
AARS2	ENSG00000124608	alanyl-tRNA synthetase 2 mitochondrial
AASS	ENSG00000008311	aminoadipate-semialdehyde synthase
ABAT	ENSG00000183044	4-aminobutyrate aminotransferase
ABCA13	ENSG00000179869	ATP binding cassette subfamily A member 13
ABCA9	ENSG00000154258	ATP binding cassette subfamily A member 9
ABCB10	ENSG00000135776	ATP binding cassette subfamily B member 10
ABCB6	ENSG00000115657	ATP binding cassette subfamily B member 6 (Langereis blood group)
ABCB7	ENSG00000131269	ATP binding cassette subfamily B member 7
ABCB8	ENSG00000197150	ATP binding cassette subfamily B member 8
ABCB9	ENSG00000150967	ATP binding cassette subfamily B member 9
ABCD1	ENSG00000101986	ATP binding cassette subfamily D member 1
ABCD2	ENSG00000173208	ATP binding cassette subfamily D member 2
ABCD3	ENSG00000117528	ATP binding cassette subfamily D member 3
ABCE1	ENSG00000164163	ATP binding cassette subfamily E member 1
ABCF2	ENSG00000033050	ATP binding cassette subfamily F member 2
ABCG1	ENSG00000160179	ATP binding cassette subfamily G member 1
ABCG2	ENSG00000118777	ATP binding cassette subfamily G member 2 (Junior blood group)
ABHD10	ENSG00000144827	abhydrolase domain containing 10 depalmitoylase
ABHD11	ENSG00000106077	abhydrolase domain containing 11
ABHD6	ENSG00000163686	abhydrolase domain containing 6 acylglycerol lipase
ABL1	ENSG00000097007	ABL proto-oncogene 1 non-receptor tyrosine kinase
ACAA1	ENSG00000060971	acetyl-CoA acyltransferase 1
ACAA2	ENSG00000167315	acetyl-CoA acyltransferase 2
ACACA	ENSG00000278540	acetyl-CoA carboxylase alpha
ACACB	ENSG00000076555	acetyl-CoA carboxylase beta
ACAD10	ENSG00000111271	acyl-CoA dehydrogenase family member 10
ACAD11	ENSG00000240303	acyl-CoA dehydrogenase family member 11
ACAD8	ENSG00000151498	acyl-CoA dehydrogenase family member 8
ACAD9	ENSG00000177646	acyl-CoA dehydrogenase family member 9
ACADL	ENSG00000115361	acyl-CoA dehydrogenase long chain
ACADM	ENSG00000117054	acyl-CoA dehydrogenase medium chain
ACADS	ENSG00000122971	acyl-CoA dehydrogenase short chain
ACADSB	ENSG00000196177	acyl-CoA dehydrogenase short/branched chain
ACADVL	ENSG00000072778	acyl-CoA dehydrogenase very long chain
ACAT1	ENSG00000075239	acetyl-CoA acetyltransferase 1
ACBD3	ENSG00000182827	acyl-CoA binding domain containing 3
ACCS	ENSG00000110455	1-aminocyclopropane-1-carboxylate synthase homolog (inactive)
ACLY	ENSG00000131473	ATP citrate lyase
ACN9	ENSG00000196636	succinate dehydrogenase complex assembly factor 3
ACO1	ENSG00000122729	aconitase 1
ACO1	ENSG00000122729	aconitase 1
ACO2	ENSG00000100412	aconitase 2
ACOT2	ENSG00000119673	acyl-CoA thioesterase 2
ACOT7	ENSG00000097021	acyl-CoA thioesterase 7
ACOT8	ENSG00000101473	acyl-CoA thioesterase 8

ACOT9	ENSG00000123130	acyl-CoA thioesterase 9
ACOX1	ENSG00000161533	acyl-CoA oxidase 1
ACOX3	ENSG00000087008	acyl-CoA oxidase 3 pristanoyl
ACP6	ENSG00000162836	acid phosphatase 6 lysophosphatidic
ACSBG2	ENSG00000130377	acyl-CoA synthetase bubblegum family member 2
ACSF2	ENSG00000167107	acyl-CoA synthetase family member 2
ACSF3	ENSG00000176715	acyl-CoA synthetase family member 3
ACSL1	ENSG00000151726	acyl-CoA synthetase long chain family member 1
ACSL4	ENSG00000068366	acyl-CoA synthetase long chain family member 4
ACSL5	ENSG00000197142	acyl-CoA synthetase long chain family member 5
ACSL6	ENSG00000164398	acyl-CoA synthetase long chain family member 6
ACSM1	ENSG00000166743	acyl-CoA synthetase medium chain family member 1
ACSM2A	ENSG00000183747	acyl-CoA synthetase medium chain family member 2A
ACSM2B	ENSG00000066813	acyl-CoA synthetase medium chain family member 2B
ACSM3	ENSG00000005187	acyl-CoA synthetase medium chain family member 3
ACSM4	ENSG00000215009	acyl-CoA synthetase medium chain family member 4
ACSM6	ENSG00000173124	acyl-CoA synthetase medium chain family member 6
ACSS1	ENSG00000154930	acyl-CoA synthetase short chain family member 1
ACSS3	ENSG00000111058	acyl-CoA synthetase short chain family member 3
ACYP2	ENSG00000170634	acylphosphatase 2
ADCK1	ENSG00000063761	aarF domain containing kinase 1
ADCK2	ENSG00000133597	aarF domain containing kinase 2
ADCK3	ENSG00000163050	coenzyme Q8A
ADCK4	ENSG00000123815	coenzyme Q8B
ADCK5	ENSG00000173137	aarF domain containing kinase 5
ADCY10	ENSG00000143199	adenylate cyclase 10
ADH5	ENSG00000197894	alcohol dehydrogenase 5 (class III) chi polypeptide
ADHFE1	ENSG00000147576	alcohol dehydrogenase iron containing 1
ADO	ENSG00000181915	2-aminoethanethiol dioxygenase
ADPRHL2	ENSG00000116863	ADP-ribosylserine hydrolase
ADSL	ENSG00000239900	adenylosuccinate lyase
AFF4	ENSG00000072364	ALF transcription elongation factor 4
AFG3L2	ENSG00000141385	AFG3 like matrix AAA peptidase subunit 2
AGAP2	ENSG00000135439	ArfGAP with GTPase domain ankyrin repeat and PH domain 2
AGK	ENSG00000006530	acylglycerol kinase
AGMAT	ENSG00000116771	agmatinase
AGPAT5	ENSG00000155189	1-acylglycerol-3-phosphate O-acyltransferase 5
AGPS	ENSG00000018510	alkylglycerone phosphate synthase
AGR2	ENSG00000106541	anterior gradient 2 protein disulphide isomerase family member
AGTPBP1	ENSG00000135049	ATP/GTP binding carboxypeptidase 1
AGXT	ENSG00000172482	alanine--glyoxylate aminotransferase
AGXT2	ENSG00000113492	alanine--glyoxylate aminotransferase 2
AHCYL1	ENSG00000168710	adenosylhomocysteinase like 1
AIFM1	ENSG00000156709	apoptosis inducing factor mitochondria associated 1
AIFM2	ENSG00000042286	apoptosis inducing factor mitochondria associated 2
AIFM3	ENSG00000183773	apoptosis inducing factor mitochondria associated 3
AK2	ENSG00000004455	adenylate kinase 2
AK3	ENSG00000147853	adenylate kinase 3
AK4	ENSG00000162433	adenylate kinase 4
AKAP1	ENSG00000121057	A-kinase anchoring protein 1
AKAP10	ENSG00000108599	A-kinase anchoring protein 10
AKAP8	ENSG00000105127	A-kinase anchoring protein 8

AKR1B10	ENSG00000198074	aldo-keto reductase family 1 member B10
AKR7A2	ENSG00000053371	aldo-keto reductase family 7 member A2
AKT1	ENSG00000142208	AKT serine/threonine kinase 1
ALAS1	ENSG00000023330	5'-aminolevulinate synthase 1
ALAS2	ENSG00000158578	5'-aminolevulinate synthase 2
ALDH18A1	ENSG00000059573	aldehyde dehydrogenase 18 family member A1
ALDH1B1	ENSG00000137124	aldehyde dehydrogenase 1 family member B1
ALDH1L1	ENSG00000144908	aldehyde dehydrogenase 1 family member L1
ALDH1L2	ENSG00000136010	aldehyde dehydrogenase 1 family member L2
ALDH2	ENSG00000111275	aldehyde dehydrogenase 2 family member
ALDH3A2	ENSG00000072210	aldehyde dehydrogenase 3 family member A2
ALDH4A1	ENSG00000159423	aldehyde dehydrogenase 4 family member A1
ALDH5A1	ENSG00000112294	aldehyde dehydrogenase 5 family member A1
ALDH6A1	ENSG00000119711	aldehyde dehydrogenase 6 family member A1
ALDH7A1	ENSG00000164904	aldehyde dehydrogenase 7 family member A1
ALDH9A1	ENSG00000143149	aldehyde dehydrogenase 9 family member A1
ALDOC	ENSG00000109107	aldolase fructose-bisphosphate C
ALKBH1	ENSG00000100601	alkB homolog 1 histone H2A dioxygenase
ALKBH3	ENSG00000166199	alkB homolog 3 alpha-ketoglutarate dependent dioxygenase
AMACR	ENSG00000242110	alpha-methylacyl-CoA racemase
AMT	ENSG00000145020	aminomethyltransferase
ANGEL2	ENSG00000174606	angel homolog 2
ANXA10	ENSG00000109511	annexin A10
AP2M1	ENSG00000161203	adaptor related protein complex 2 subunit mu 1
APEX1	ENSG00000100823	apurinic/apyrimidinic endodeoxyribonuclease 1
APEX2	ENSG00000169188	apurinic/apyrimidinic endodeoxyribonuclease 2
APOA1BP	ENSG00000163382	NAD(P)HX epimerase
APOO	ENSG00000184831	apolipoprotein O
APOOL	ENSG00000155008	apolipoprotein O like
APOPT1	ENSG00000256053	cytochrome c oxidase assembly factor 8
ARAF	ENSG00000078061	A-Raf proto-oncogene serine/threonine kinase
ARF5	ENSG00000004059	ADP ribosylation factor 5
ARG2	ENSG00000081181	arginase 2
ARGLU1	ENSG00000134884	arginine and glutamate rich 1
ARL2	ENSG00000213465	ADP ribosylation factor like GTPase 2
ARMC1	ENSG00000104442	armadillo repeat containing 1
ARMC10	ENSG00000170632	armadillo repeat containing 10
ARMS2	ENSG00000254636	age-related maculopathy susceptibility 2
ARSB	ENSG00000113273	arylsulfatase B
AS3MT	ENSG00000214435	arsenite methyltransferase
ASAH2	ENSG00000188611	N-acylsphingosine amidohydrolase 2
ASB9	ENSG00000102048	ankyrin repeat and SOCS box containing 9
ATAD1	ENSG00000138138	ATPase family AAA domain containing 1
ATAD3A	ENSG00000197785	ATPase family AAA domain containing 3A
ATAD3B	ENSG00000160072	ATPase family AAA domain containing 3B
ATCAY	ENSG00000167654	ATCAY kinesin light chain interacting caytaxin
ATG4D	ENSG00000130734	autophagy related 4D cysteine peptidase
ATIC	ENSG00000138363	5-aminoimidazole-4-carboxamide ribonucleotide formyltransferase/IMP cyclohydrolase
ATP10D	ENSG00000145246	ATPase phospholipid transporting 10D (putative)
ATP5A1	ENSG00000152234	ATP synthase F1 subunit alpha
ATP5B	ENSG00000110955	ATP synthase F1 subunit beta

ATP5C1	ENSG00000165629	ATP synthase F1 subunit gamma
ATP5D	ENSG00000099624	ATP synthase F1 subunit delta
ATP5EP2	ENSG00000180389	ATP synthase F1 subunit epsilon pseudogene 2
ATP5F1	ENSG00000116459	ATP synthase peripheral stalk-membrane subunit b
ATP5G1	ENSG00000159199	ATP synthase membrane subunit c locus 1
ATP5G2	ENSG00000135390	ATP synthase membrane subunit c locus 2
ATP5G3	ENSG00000154518	ATP synthase membrane subunit c locus 3
ATP5H	ENSG00000167863	ATP synthase peripheral stalk subunit d
ATP5I	ENSG00000169020	ATP synthase membrane subunit e
ATP5J	ENSG00000154723	ATP synthase peripheral stalk subunit F6
ATP5J2-PTCD1	ENSG00000248919	ATP5MF-PTCD1 readthrough
ATP5L	ENSG00000167283	ATP synthase membrane subunit g
ATP5O	ENSG00000241837	ATP synthase peripheral stalk subunit OSCP
ATP5S	ENSG00000125375	distal membrane arm assembly component 2 like
ATP5SL	ENSG00000105341	distal membrane arm assembly component 2
ATP6V1A	ENSG00000114573	ATPase H+ transporting V1 subunit A
ATP6V1E1	ENSG00000131100	ATPase H+ transporting V1 subunit E1
ATP7B	ENSG00000123191	ATPase copper transporting beta
ATPAF1	ENSG00000123472	ATP synthase mitochondrial F1 complex assembly factor 1
ATPAF2	ENSG00000171953	ATP synthase mitochondrial F1 complex assembly factor 2
ATPIF1	ENSG00000130770	ATP synthase inhibitory factor subunit 1
ATRX	ENSG00000085224	ATRX chromatin remodeler
ATXN2	ENSG00000204842	ataxin 2
AUH	ENSG00000148090	AU RNA binding methylglutaconyl-CoA hydratase
AZIN2	ENSG00000142920	antizyme inhibitor 2
BAD	ENSG00000002330	BCL2 associated agonist of cell death
BAG5	ENSG00000166170	BAG cochaperone 5
BAK1	ENSG00000030110	BCL2 antagonist/killer 1
BARX2	ENSG00000043039	BARX homeobox 2
BAX	ENSG00000087088	BCL2 associated X apoptosis regulator
BBC3	ENSG00000105327	BCL2 binding component 3
BBOX1	ENSG00000129151	gamma-butyrobetaine hydroxylase 1
BCAT1	ENSG00000060982	branched chain amino acid transaminase 1
BCAT2	ENSG00000105552	branched chain amino acid transaminase 2
BCKDHA	ENSG00000248098	branched chain keto acid dehydrogenase E1 subunit alpha
BCKDHB	ENSG00000083123	branched chain keto acid dehydrogenase E1 subunit beta
BCKDK	ENSG00000103507	branched chain keto acid dehydrogenase kinase
BCL2	ENSG00000171791	BCL2 apoptosis regulator
BCL2L1	ENSG00000171552	BCL2 like 1
BCL2L10	ENSG00000137875	BCL2 like 10
BCL2L11	ENSG00000153094	BCL2 like 11
BCL2L13	ENSG00000099968	BCL2 like 13
BCL2L2	ENSG00000129473	BCL2 like 2
BCLAF3	ENSG00000173681	BCLAF1 and THRAP3 family member 3
BCO2	ENSG00000197580	beta-carotene oxygenase 2
BCS1L	ENSG00000074582	BCS1 homolog ubiquinol-cytochrome c reductase complex chaperone
BDH1	ENSG00000161267	3-hydroxybutyrate dehydrogenase 1
BDH2	ENSG00000164039	3-hydroxybutyrate dehydrogenase 2
BFSP1	ENSG00000125864	beaded filament structural protein 1
BID	ENSG00000015475	BH3 interacting domain death agonist
BLID	ENSG00000259571	BH3-like motif containing cell death inducer

BLOC1S1	ENSG00000135441	biogenesis of lysosomal organelles complex 1 subunit 1
BLOC1S2	ENSG00000196072	biogenesis of lysosomal organelles complex 1 subunit 2
BNIP1	ENSG00000113734	BCL2 interacting protein 1
BNIP3	ENSG00000176171	BCL2 interacting protein 3
BNIP3L	ENSG00000104765	BCL2 interacting protein 3 like
BOK	ENSG00000176720	BCL2 family apoptosis regulator BOK
BOLA1	ENSG00000178096	bolA family member 1
BPHL	ENSG00000137274	biphenyl hydrolase like
BRAF	ENSG00000157764	B-Raf proto-oncogene serine/threonine kinase
BRD8	ENSG00000112983	bromodomain containing 8
BRINP3	ENSG00000162670	BMP/retinoic acid inducible neural specific 3
BSG	ENSG00000172270	basigin (Ok blood group)
BZRAP1	ENSG00000005379	TSPO associated protein 1
C10orf10	ENSG00000165507	DEPP autophagy regulator 1
C10orf2	ENSG00000107815	twinkle mtDNA helicase
C12orf10	ENSG00000139637	MYG1 exonuclease
C12orf65	ENSG00000130921	mitochondrial translation release factor in rescue
C14orf119	ENSG00000179933	chromosome 14 open reading frame 119
C14orf159	ENSG00000133943	D-glutamate cyclase
C14orf2	ENSG00000156411	ATP synthase membrane subunit j
C15orf40	ENSG00000169609	chromosome 15 open reading frame 40
C15orf48	ENSG00000166920	chromosome 15 open reading frame 48
C15orf48	ENSG00000166920	chromosome 15 open reading frame 48
C15orf61	ENSG00000189227	chromosome 15 open reading frame 61
C15orf62	ENSG00000188277	chromosome 15 open reading frame 62
C16orf91	ENSG00000174109	chromosome 16 open reading frame 91
C17orf89	ENSG00000224877	NADH:ubiquinone oxidoreductase complex assembly factor 8
C19orf12	ENSG00000131943	chromosome 19 open reading frame 12
C19orf52	ENSG00000142444	translocase of inner mitochondrial membrane 29
C19orf70	ENSG00000174917	mitochondrial contact site and cristae organizing system subunit 13
C1QBP	ENSG00000108561	complement C1q binding protein
C20orf24	ENSG00000101084	RAB5 interacting factor
C21orf33	ENSG00000160221	glutamine amidotransferase class 1 domain containing 3
C2orf47	ENSG00000162972	matrix AAA peptidase interacting protein 1
C2orf69	ENSG00000178074	chromosome 2 open reading frame 69
C3orf33	ENSG00000174928	chromosome 3 open reading frame 33
C5orf63	ENSG00000164241	chromosome 5 open reading frame 63
C6orf136	ENSG00000204564	chromosome 6 open reading frame 136
C6orf203	ENSG00000130349	mitochondrial transcription rescue factor 1
C6orf57	ENSG00000154079	succinate dehydrogenase complex assembly factor 4
C7orf55	ENSG00000164898	formation of mitochondrial complex V assembly factor 1 homolog
C8orf82	ENSG00000213563	chromosome 8 open reading frame 82
CA5A	ENSG00000174990	carbonic anhydrase 5A
CA5B	ENSG00000169239	carbonic anhydrase 5B
CA5BP1	ENSG00000186312	carbonic anhydrase 5B pseudogene 1
CAMK2A	ENSG00000070808	calcium/calmodulin dependent protein kinase II alpha
CAPN10	ENSG00000142330	calpain 10
CAPRIN2	ENSG00000110888	caprin family member 2
CARD19	ENSG00000165233	caspase recruitment domain family member 19
CARKD	ENSG00000213995	NAD(P)HX dehydratase
CARS2	ENSG00000134905	cysteinyl-tRNA synthetase 2 mitochondrial
CASP2	ENSG00000106144	caspase 2

CASP4	ENSG00000196954	caspase 4
CASP8	ENSG00000064012	caspase 8
CASP8AP2	ENSG00000118412	caspase 8 associated protein 2
CASP9	ENSG00000132906	caspase 9
CASQ1	ENSG00000143318	calsequestrin 1
CAT	ENSG00000121691	catalase
CBR3	ENSG00000159231	carbonyl reductase 3
CBR4	ENSG00000145439	carbonyl reductase 4
CCBL2	ENSG00000137944	kynurenine aminotransferase 3
CCDC109B	ENSG00000005059	mitochondrial calcium uniporter dominant negative subunit beta
CCDC127	ENSG00000164366	coiled-coil domain containing 127
CCDC136	ENSG00000128596	coiled-coil domain containing 136
CCDC19	ENSG00000213085	cilia and flagella associated protein 45
CCDC51	ENSG00000164051	coiled-coil domain containing 51
CCDC58	ENSG00000160124	mitochondrial matrix import factor 23
CCDC90B	ENSG00000137500	coiled-coil domain containing 90B
CCT7	ENSG00000135624	chaperonin containing TCP1 subunit 7
CD3EAP	ENSG00000117877	RNA polymerase I subunit G
CDC25C	ENSG00000158402	cell division cycle 25C
CDK1	ENSG00000170312	cyclin dependent kinase 1
CDK7	ENSG00000134058	cyclin dependent kinase 7
CDKN2A	ENSG00000147889	cyclin dependent kinase inhibitor 2A
CECR5	ENSG00000069998	haloacid dehalogenase like hydrolase domain containing 5
CEP89	ENSG00000121289	centrosomal protein 89
CERK	ENSG00000100422	ceramide kinase
CHAT	ENSG00000070748	choline O-acetyltransferase
CHCHD1	ENSG00000172586	coiled-coil-helix-coiled-coil-helix domain containing 1
CHCHD10	ENSG00000250479	coiled-coil-helix-coiled-coil-helix domain containing 10
CHCHD2P9	ENSG00000186940	coiled-coil-helix-coiled-coil-helix domain containing 2 pseudogene 9
CHCHD3	ENSG00000106554	coiled-coil-helix-coiled-coil-helix domain containing 3
CHCHD4	ENSG00000163528	coiled-coil-helix-coiled-coil-helix domain containing 4
CHCHD5	ENSG00000125611	coiled-coil-helix-coiled-coil-helix domain containing 5
CHCHD6	ENSG00000159685	coiled-coil-helix-coiled-coil-helix domain containing 6
CHCHD7	ENSG00000170791	coiled-coil-helix-coiled-coil-helix domain containing 7
CHDH	ENSG00000016391	choline dehydrogenase
CHMP2B	ENSG00000083937	charged multivesicular body protein 2B
CHPF	ENSG00000123989	chondroitin polymerizing factor
CHPT1	ENSG00000111666	choline phosphotransferase 1
CIAPIN1	ENSG00000005194	cytokine induced apoptosis inhibitor 1
CIDEA	ENSG00000176194	cell death inducing DFFA like effector a
CISD1	ENSG00000122873	CDGSH iron sulfur domain 1
CISD2	ENSG00000145354	CDGSH iron sulfur domain 2
CISD3	ENSG00000277972	CDGSH iron sulfur domain 3
CKB	ENSG00000166165	creatine kinase B
CKMT1B	ENSG00000237289	creatine kinase mitochondrial 1B
CKMT2	ENSG00000131730	creatine kinase mitochondrial 2
CLIC1	ENSG00000213719	chloride intracellular channel 1
CLIC4	ENSG00000169504	chloride intracellular channel 4
CLN3	ENSG00000188603	CLN3 lysosomal/endosomal transmembrane protein battenin
CLN8	ENSG00000182372	CLN8 transmembrane ER and ERGIC protein
CLPB	ENSG00000162129	caseinolytic mitochondrial matrix peptidase chaperone subunit B
CLPP	ENSG00000125656	caseinolytic mitochondrial matrix peptidase proteolytic subunit

CLPX	ENSG00000166855	caseinolytic mitochondrial matrix peptidase chaperone subunit X
CLTC	ENSG00000141367	clathrin heavy chain
CLU	ENSG00000120885	clusterin
CLYBL	ENSG00000125246	citramalyl-CoA lyase
CMC4	ENSG00000182712	C-X9-C motif containing 4
COA4	ENSG00000181924	cytochrome c oxidase assembly factor 4 homolog
COA7	ENSG00000162377	cytochrome c oxidase assembly factor 7
COASY	ENSG00000068120	Coenzyme A synthase
COL4A3BP	ENSG00000113163	ceramide transporter 1
COMT	ENSG00000093010	catechol-O-methyltransferase
COMTD1	ENSG00000165644	catechol-O-methyltransferase domain containing 1
COQ10A	ENSG00000135469	coenzyme Q10A
COQ10B	ENSG00000115520	coenzyme Q10B
COQ2	ENSG00000173085	coenzyme Q2 polyprenyltransferase
COQ3	ENSG00000132423	coenzyme Q3 methyltransferase
COQ4	ENSG00000167113	coenzyme Q4
COQ5	ENSG00000110871	coenzyme Q5 methyltransferase
COQ6	ENSG00000119723	coenzyme Q6 monooxygenase
COQ7	ENSG00000167186	coenzyme Q7 hydroxylase
COQ9	ENSG00000088682	coenzyme Q9
COX10	ENSG00000006695	cytochrome c oxidase assembly factor heme A:farnesyltransferase COX10
COX14	ENSG00000178449	cytochrome c oxidase assembly factor COX14
COX15	ENSG00000014919	cytochrome c oxidase assembly homolog COX15
COX16	ENSG00000133983	cytochrome c oxidase assembly factor COX16
COX17	ENSG00000138495	cytochrome c oxidase copper chaperone COX17
COX18	ENSG00000163626	cytochrome c oxidase assembly factor COX18
COX19	ENSG00000240230	cytochrome c oxidase assembly factor COX19
COX20	ENSG00000203667	cytochrome c oxidase assembly factor COX20
COX4I1	ENSG00000131143	cytochrome c oxidase subunit 4I1
COX4I2	ENSG00000131055	cytochrome c oxidase subunit 4I2
COX5A	ENSG00000178741	cytochrome c oxidase subunit 5A
COX5B	ENSG00000135940	cytochrome c oxidase subunit 5B
COX6A1	ENSG00000111775	cytochrome c oxidase subunit 6A1
COX6A2	ENSG00000156885	cytochrome c oxidase subunit 6A2
COX6B1	ENSG00000126267	cytochrome c oxidase subunit 6B1
COX6B2	ENSG00000160471	cytochrome c oxidase subunit 6B2
COX6C	ENSG00000164919	cytochrome c oxidase subunit 6C
COX7A1	ENSG00000161281	cytochrome c oxidase subunit 7A1
COX7A2	ENSG00000112695	cytochrome c oxidase subunit 7A2
COX7A2L	ENSG00000115944	cytochrome c oxidase subunit 7A2 like
COX7B	ENSG00000131174	cytochrome c oxidase subunit 7B
COX7C	ENSG00000127184	cytochrome c oxidase subunit 7C
COX8A	ENSG00000176340	cytochrome c oxidase subunit 8A
COX8C	ENSG00000187581	cytochrome c oxidase subunit 8C
CPOX	ENSG00000080819	coproporphyrinogen oxidase
CPS1	ENSG00000021826	carbamoyl-phosphate synthase 1
CPT1A	ENSG00000110090	carnitine palmitoyltransferase 1A
CPT1B	ENSG00000205560	carnitine palmitoyltransferase 1B
CPT1C	ENSG00000169169	carnitine palmitoyltransferase 1C
CPT2	ENSG00000157184	carnitine palmitoyltransferase 2
CRAT	ENSG00000095321	carnitine O-acetyltransferase

CRLS1	ENSG00000088766	cardiolipin synthase 1
CROT	ENSG00000005469	carnitine O-octanoyltransferase
CRY1	ENSG00000008405	cryptochrome circadian regulator 1
CRY2	ENSG00000121671	cryptochrome circadian regulator 2
CRYAB	ENSG00000109846	crystallin alpha B
CRYM	ENSG00000103316	crystallin mu
CRYZ	ENSG00000116791	crystallin zeta
CS	ENSG00000062485	citrate synthase
CTPS2	ENSG00000047230	CTP synthase 2
CTSA	ENSG00000064601	cathepsin A
CTSB	ENSG00000164733	cathepsin B
CTSD	ENSG00000117984	cathepsin D
CTU1	ENSG00000142544	cytosolic thiouridylase subunit 1
CYB5A	ENSG00000166347	cytochrome b5 type A
CYB5B	ENSG00000103018	cytochrome b5 type B
CYB5R1	ENSG00000159348	cytochrome b5 reductase 1
CYB5R2	ENSG00000166394	cytochrome b5 reductase 2
CYB5R3	ENSG00000100243	cytochrome b5 reductase 3
CYBA	ENSG00000051523	cytochrome b-245 alpha chain
CYBB	ENSG00000165168	cytochrome b-245 beta chain
CYC1	ENSG00000179091	cytochrome c1
CYCS	ENSG00000172115	cytochrome c somatic
CYP11A1	ENSG00000140459	cytochrome P450 family 11 subfamily A member 1
CYP11B1	ENSG00000160882	cytochrome P450 family 11 subfamily B member 1
CYP11B2	ENSG00000179142	cytochrome P450 family 11 subfamily B member 2
CYP17A1	ENSG00000148795	cytochrome P450 family 17 subfamily A member 1
CYP1A1	ENSG00000140465	cytochrome P450 family 1 subfamily A member 1
CYP1B1	ENSG00000138061	cytochrome P450 family 1 subfamily B member 1
CYP24A1	ENSG00000019186	cytochrome P450 family 24 subfamily A member 1
CYP27A1	ENSG00000135929	cytochrome P450 family 27 subfamily A member 1
CYP27B1	ENSG00000111012	cytochrome P450 family 27 subfamily B member 1
CYP2D6	ENSG00000100197	cytochrome P450 family 2 subfamily D member 6
CYP2E1	ENSG00000130649	cytochrome P450 family 2 subfamily E member 1
D2HGDH	ENSG00000180902	D-2-hydroxyglutarate dehydrogenase
DACT2	ENSG00000164488	dishevelled binding antagonist of beta catenin 2
DAOA	ENSG00000182346	D-amino acid oxidase activator
DAP3	ENSG00000132676	death associated protein 3
DARS2	ENSG00000117593	aspartyl-tRNA synthetase 2 mitochondrial
DBI	ENSG00000155368	diazepam binding inhibitor acyl-CoA binding protein
DBT	ENSG00000137992	dihydrolipoamide branched chain transacylase E2
DCAF5	ENSG00000139990	DDB1 and CUL4 associated factor 5
DCAKD	ENSG00000172992	dephospho-CoA kinase domain containing
DCPS	ENSG00000110063	decapping enzyme scavenger
DCXR	ENSG00000169738	dicarbonyl and L-xylulose reductase
DDAH1	ENSG00000153904	dimethylarginine dimethylaminohydrolase 1
DDAH2	ENSG00000213722	dimethylarginine dimethylaminohydrolase 2
DDHD2	ENSG00000085788	DDHD domain containing 2
DDIT4	ENSG00000168209	DNA damage inducible transcript 4
DDX23	ENSG00000174243	DEAD-box helicase 23
DDX28	ENSG00000182810	DEAD-box helicase 28
DECR1	ENSG00000104325	2 4-dienoyl-CoA reductase 1
DEPP1	ENSG00000165507	DEPP autophagy regulator 1

DGAT2	ENSG00000062282	diacylglycerol O-acyltransferase 2
DGUOK	ENSG00000114956	deoxyguanosine kinase
DHCR24	ENSG00000116133	24-dehydrocholesterol reductase
DHFRL1	ENSG00000178700	dihydrofolate reductase 2
DHODH	ENSG00000102967	dihydroorotate dehydrogenase (quinone)
DHRS1	ENSG00000157379	dehydrogenase/reductase 1
DHRS2	ENSG00000100867	dehydrogenase/reductase 2
DHRS4	ENSG00000157326	dehydrogenase/reductase 4
DHRS7B	ENSG00000109016	dehydrogenase/reductase 7B
DHRSX	ENSG00000169084	dehydrogenase/reductase X-linked
DHTKD1	ENSG00000181192	dehydrogenase E1 and transketolase domain containing 1
DHX29	ENSG00000067248	DExH-box helicase 29
DHX30	ENSG00000132153	DExH-box helicase 30
DHX32	ENSG00000089876	DEAH-box helicase 32 (putative)
DIABLO	ENSG00000184047	diablo IAP-binding mitochondrial protein
DISC1	ENSG00000162946	DISC1 scaffold protein
DLAT	ENSG00000150768	dihydrolipoamide S-acetyltransferase
DLD	ENSG00000091140	dihydrolipoamide dehydrogenase
DLST	ENSG00000119689	dihydrolipoamide S-succinyltransferase
DMGDH	ENSG00000132837	dimethylglycine dehydrogenase
DMPK	ENSG00000104936	DM1 protein kinase
DNA2	ENSG00000138346	DNA replication helicase/nuclease 2
DNAJA1	ENSG00000086061	DnaJ heat shock protein family (Hsp40) member A1
DNAJA3	ENSG00000103423	DnaJ heat shock protein family (Hsp40) member A3
DNAJC11	ENSG00000007923	DnaJ heat shock protein family (Hsp40) member C11
DNAJC15	ENSG00000120675	DnaJ heat shock protein family (Hsp40) member C15
DNAJC19	ENSG00000205981	DnaJ heat shock protein family (Hsp40) member C19
DNAJC27	ENSG00000115137	DnaJ heat shock protein family (Hsp40) member C27
DNAJC28	ENSG00000177692	DnaJ heat shock protein family (Hsp40) member C28
DNAJC30	ENSG00000176410	DnaJ heat shock protein family (Hsp40) member C30
DNAJC4	ENSG00000110011	DnaJ heat shock protein family (Hsp40) member C4
DNAJC5	ENSG00000101152	DnaJ heat shock protein family (Hsp40) member C5
DNLZ	ENSG00000213221	DNL-type zinc finger
DNM1L	ENSG00000087470	dynamin 1 like
DNM3	ENSG00000197959	dynamin 3
DPYSL2	ENSG00000092964	dihydropyrimidinase like 2
DRG2	ENSG00000108591	developmentally regulated GTP binding protein 2
DSP	ENSG00000096696	desmoplakin
DUS2	ENSG00000167264	dihydrouridine synthase 2
DUSP26	ENSG00000133878	dual specificity phosphatase 26
DUT	ENSG00000128951	deoxyuridine triphosphatase
E9PI62	ENSG00000254536	
EARS2	ENSG00000103356	glutamyl-tRNA synthetase 2 mitochondrial
ECH1	ENSG00000104823	enoyl-CoA hydratase 1
ECHDC1	ENSG00000093144	ethylmalonyl-CoA decarboxylase 1
ECHDC2	ENSG00000121310	enoyl-CoA hydratase domain containing 2
ECHDC3	ENSG00000134463	enoyl-CoA hydratase domain containing 3
ECHS1	ENSG00000127884	enoyl-CoA hydratase short chain 1
ECI1	ENSG00000167969	enoyl-CoA delta isomerase 1
ECI2	ENSG00000198721	enoyl-CoA delta isomerase 2
ECSIT	ENSG00000130159	ECSIT signaling integrator
EEFSEC	ENSG00000132394	eukaryotic elongation factor selenocysteine-tRNA specific

EFHD1	ENSG00000115468	EF-hand domain family member D1
EHHADH	ENSG00000113790	enoyl-CoA hydratase and 3-hydroxyacyl CoA dehydrogenase
ELAC2	ENSG00000006744	elaC ribonuclease Z 2
ELK3	ENSG00000111145	ETS transcription factor ELK3
EMC8	ENSG00000131148	ER membrane protein complex subunit 8
ENDOG	ENSG00000167136	endonuclease G
ENOSF1	ENSG00000132199	enolase superfamily member 1
EPHX2	ENSG00000120915	epoxide hydrolase 2
ERAL1	ENSG00000132591	Era like 12S mitochondrial rRNA chaperone 1
ERCC6L2	ENSG00000182150	ERCC excision repair 6 like 2
ERN1	ENSG00000178607	endoplasmic reticulum to nucleus signaling 1
ESR2	ENSG00000140009	estrogen receptor 2
ETFA	ENSG00000140374	electron transfer flavoprotein subunit alpha
ETFB	ENSG00000105379	electron transfer flavoprotein subunit beta
ETFDH	ENSG00000171503	electron transfer flavoprotein dehydrogenase
ETNPPL	ENSG00000164089	ethanolamine-phosphate phospho-lyase
EXOG	ENSG00000157036	exo/endonuclease G
F5H5T6	ENSG00000256591	
FABP1	ENSG00000163586	fatty acid binding protein 1
FADS1	ENSG00000149485	fatty acid desaturase 1
FAHD1	ENSG00000180185	fumarylacetoacetate hydrolase domain containing 1
FAHD2A	ENSG00000115042	fumarylacetoacetate hydrolase domain containing 2A
FAM136A	ENSG00000035141	family with sequence similarity 136 member A
FAM162A	ENSG00000114023	family with sequence similarity 162 member A
FAM185A	ENSG00000222011	family with sequence similarity 185 member A
FAM213A	ENSG00000122378	peroxiredoxin like 2A
FAM32A	ENSG00000105058	family with sequence similarity 32 member A
FAM65B	ENSG00000111913	RHO family interacting cell polarization regulator 2
FAM72A	ENSG00000196550	family with sequence similarity 72 member A
FANCG	ENSG00000221829	FA complementation group G
FARS2	ENSG00000145982	phenylalanyl-tRNA synthetase 2 mitochondrial
FASN	ENSG00000169710	fatty acid synthase
FASTK	ENSG00000164896	Fas activated serine/threonine kinase
FASTKD1	ENSG00000138399	FAST kinase domains 1
FASTKD2	ENSG00000118246	FAST kinase domains 2
FASTKD3	ENSG00000124279	FAST kinase domains 3
FASTKD5	ENSG00000215251	FAST kinase domains 5
FBXL4	ENSG00000112234	F-box and leucine rich repeat protein 4
FBXO7	ENSG00000100225	F-box protein 7
FDPS	ENSG00000160752	farnesyl diphosphate synthase
FDX1	ENSG00000137714	ferredoxin 1
FDX1L	ENSG00000267673	ferredoxin 2
FDXR	ENSG00000161513	ferredoxin reductase
FECH	ENSG00000066926	ferrochelatase
FEN1	ENSG00000168496	flap structure-specific endonuclease 1
FH	ENSG00000091483	fumarate hydratase
FHIT	ENSG00000189283	fragile histidine triad diadenosine triphosphatase
FIBP	ENSG00000172500	FGF1 intracellular binding protein
FIS1	ENSG00000214253	fission mitochondrial 1
FITM2	ENSG00000197296	fat storage inducing transmembrane protein 2
FKBP10	ENSG00000141756	FKBP prolyl isomerase 10
FKBP4	ENSG00000004478	FKBP prolyl isomerase 4

FKBP8	ENSG00000105701	FKBP prolyl isomerase 8
FLAD1	ENSG00000160688	flavin adenine dinucleotide synthetase 1
FLVCR1	ENSG00000162769	FLVCR heme transporter 1
FOXO1	ENSG00000150907	forkhead box O1
FOXRED1	ENSG00000110074	FAD dependent oxidoreductase domain containing 1
FPGS	ENSG00000136877	folylpolyglutamate synthase
FSIP2	ENSG00000188738	fibrous sheath interacting protein 2
FTH1	ENSG00000167996	ferritin heavy chain 1
FTMT	ENSG00000181867	ferritin mitochondrial
FTSJ2	ENSG00000122687	mitochondrial rRNA methyltransferase 2
FUNDC1	ENSG00000069509	FUN14 domain containing 1
FUNDC2	ENSG00000165775	FUN14 domain containing 2
FXN	ENSG00000165060	frataxin
FYN	ENSG00000010810	FYN proto-oncogene Src family tyrosine kinase
G0S2	ENSG00000123689	G0/G1 switch 2
GADD45GIP1	ENSG00000179271	GADD45G interacting protein 1
GAPDH	ENSG00000111640	glyceraldehyde-3-phosphate dehydrogenase
GARS	ENSG00000106105	glycyl-tRNA synthetase 1
GATC	ENSG00000257218	glutamyl-tRNA amidotransferase subunit C
GATM	ENSG00000171766	glycine amidinotransferase
GBAS	ENSG00000146729	nipsnap homolog 2
GBF1	ENSG00000107862	golgi brefeldin A resistant guanine nucleotide exchange factor 1
GCAT	ENSG00000100116	glycine C-acetyltransferase
GCDH	ENSG00000105607	glutaryl-CoA dehydrogenase
GCK	ENSG00000106633	glucokinase
GCKR	ENSG00000084734	glucokinase regulator
GCSH	ENSG00000140905	glycine cleavage system protein H
GDF5OS	ENSG00000204183	GDF5 antisense RNA 1
GFER	ENSG00000127554	growth factor augmenter of liver regeneration
GFM1	ENSG00000168827	G elongation factor mitochondrial 1
GFM2	ENSG00000164347	GTP dependent ribosome recycling factor mitochondrial 2
GHITM	ENSG00000165678	growth hormone inducible transmembrane protein
GHR	ENSG00000112964	growth hormone receptor
GIMAP8	ENSG00000171115	GTPase IMAP family member 8
GIT1	ENSG00000108262	GIT ArfGAP 1
GK	ENSG00000198814	glycerol kinase
GLDC	ENSG00000178445	glycine decarboxylase
GLOD4	ENSG00000167699	glyoxalase domain containing 4
GLRX	ENSG00000173221	glutaredoxin
GLRX2	ENSG00000023572	glutaredoxin 2
GLRX5	ENSG00000182512	glutaredoxin 5
GLS	ENSG00000115419	glutaminase
GLS2	ENSG00000135423	glutaminase 2
GLUD1	ENSG00000148672	glutamate dehydrogenase 1
GLUD2	ENSG00000182890	glutamate dehydrogenase 2
GLUL	ENSG00000135821	glutamate-ammonia ligase
GLYAT	ENSG00000149124	glycine-N-acyltransferase
GLYATL1	ENSG00000166840	glycine-N-acyltransferase like 1
GLYATL2	ENSG00000156689	glycine-N-acyltransferase like 2
GLYATL3	ENSG00000203972	glycine-N-acyltransferase like 3
GLYCTK	ENSG00000168237	glycerate kinase
GM2A	ENSG00000196743	ganglioside GM2 activator

GML	ENSG00000104499	glycosylphosphatidylinositol anchored molecule like
GMPPB	ENSG00000173540	GDP-mannose pyrophosphorylase B
GNB2L1	ENSG00000204628	receptor for activated C kinase 1
GNG5	ENSG00000174021	G protein subunit gamma 5
GNL3L	ENSG00000130119	G protein nucleolar 3 like
GNPAT	ENSG00000116906	glyceronephosphate O-acyltransferase
GOLPH3	ENSG00000113384	golgi phosphoprotein 3
GOT2	ENSG00000125166	glutamic-oxaloacetic transaminase 2
GPAM	ENSG00000119927	glycerol-3-phosphate acyltransferase mitochondrial
GPAT2	ENSG00000186281	glycerol-3-phosphate acyltransferase 2 mitochondrial
GPD1	ENSG00000167588	glycerol-3-phosphate dehydrogenase 1
GPD2	ENSG00000115159	glycerol-3-phosphate dehydrogenase 2
GPI	ENSG00000105220	glucose-6-phosphate isomerase
GPRC5C	ENSG00000170412	G protein-coupled receptor class C group 5 member C
GPT2	ENSG00000166123	glutamic--pyruvic transaminase 2
GPX1	ENSG00000233276	glutathione peroxidase 1
GPX4	ENSG00000167468	glutathione peroxidase 4
GRAMD4	ENSG00000075240	GRAM domain containing 4
GRHPR	ENSG00000137106	glyoxylate and hydroxypyruvate reductase
GRN	ENSG00000030582	granulin precursor
GRPEL1	ENSG00000109519	GrpE like 1 mitochondrial
GRSF1	ENSG00000132463	G-rich RNA sequence binding factor 1
GSDMC	ENSG00000147697	gasdermin C
GSR	ENSG00000104687	glutathione-disulfide reductase
GSTK1	ENSG00000197448	glutathione S-transferase kappa 1
GSTO1	ENSG00000148834	glutathione S-transferase omega 1
GSTP1	ENSG00000084207	glutathione S-transferase pi 1
GSTZ1	ENSG00000100577	glutathione S-transferase zeta 1
GTPBP10	ENSG00000105793	GTP binding protein 10
GTPBP3	ENSG00000130299	GTP binding protein 3 mitochondrial
GTPBP6	ENSG00000178605	GTP binding protein 6 (putative)
GTPBP8	ENSG00000163607	GTP binding protein 8 (putative)
GUF1	ENSG00000151806	GTP binding elongation factor GUF1
GUK1	ENSG00000143774	guanylate kinase 1
H6PD	ENSG00000049239	hexose-6-phosphate dehydrogenase/glucose 1-dehydrogenase
HADH	ENSG00000138796	hydroxyacyl-CoA dehydrogenase
HADHA	ENSG00000084754	hydroxyacyl-CoA dehydrogenase trifunctional multienzyme complex subunit alpha
HADHB	ENSG00000138029	hydroxyacyl-CoA dehydrogenase trifunctional multienzyme complex subunit beta
HAGH	ENSG00000063854	hydroxyacylglutathione hydrolase
HAO2	ENSG00000116882	hydroxyacid oxidase 2
HAP1	ENSG00000173805	huntingtin associated protein 1
HARS2	ENSG00000112855	histidyl-tRNA synthetase 2 mitochondrial
HAX1	ENSG00000143575	HCLS1 associated protein X-1
HCCS	ENSG00000004961	holocytochrome c synthase
HCFC1	ENSG00000172534	host cell factor C1
HCLS1	ENSG00000180353	hematopoietic cell-specific Lyn substrate 1
HDDC2	ENSG00000111906	HD domain containing 2
HDHD3	ENSG00000119431	haloacid dehalogenase like hydrolase domain containing 3
HEATR1	ENSG00000119285	HEAT repeat containing 1
HEBP1	ENSG00000013583	heme binding protein 1

HEBP2	ENSG00000051620	heme binding protein 2
HEMK1	ENSG00000114735	HemK methyltransferase family member 1
HIBADH	ENSG00000106049	3-hydroxyisobutyrate dehydrogenase
HIBCH	ENSG00000198130	3-hydroxyisobutyryl-CoA hydrolase
HIGD1A	ENSG00000181061	HIG1 hypoxia inducible domain family member 1A
HIGD2A	ENSG00000146066	HIG1 hypoxia inducible domain family member 2A
HINT1	ENSG00000169567	histidine triad nucleotide binding protein 1
HINT2	ENSG00000137133	histidine triad nucleotide binding protein 2
HINT3	ENSG00000111911	histidine triad nucleotide binding protein 3
HIVEP1	ENSG00000095951	HIVEP zinc finger 1
HK1	ENSG00000156515	hexokinase 1
HK2	ENSG00000159399	hexokinase 2
HK3	ENSG00000160883	hexokinase 3
HLCS	ENSG00000159267	holocarboxylase synthetase
HMBS	ENSG00000256269	hydroxymethylbilane synthase
HMGCL	ENSG00000117305	3-hydroxy-3-methylglutaryl-CoA lyase
HMGCS2	ENSG00000134240	3-hydroxy-3-methylglutaryl-CoA synthase 2
HOGA1	ENSG00000241935	4-hydroxy-2-oxoglutarate aldolase 1
HOXB9	ENSG00000170689	homeobox B9
HRK	ENSG00000135116	harakiri BCL2 interacting protein
HRSP12	ENSG00000132541	reactive intermediate imine deaminase A homolog
HSCB	ENSG00000100209	HscB mitochondrial iron-sulfur cluster cochaperone
HSD17B10	ENSG00000072506	hydroxysteroid 17-beta dehydrogenase 10
HSD17B4	ENSG00000133835	hydroxysteroid 17-beta dehydrogenase 4
HSD17B8	ENSG00000204228	hydroxysteroid 17-beta dehydrogenase 8
HSDL1	ENSG00000103160	hydroxysteroid dehydrogenase like 1
HSDL2	ENSG00000119471	hydroxysteroid dehydrogenase like 2
HSH2D	ENSG00000196684	hematopoietic SH2 domain containing
HSP90AA1	ENSG00000080824	heat shock protein 90 alpha family class A member 1
HSP90AB1	ENSG00000096384	heat shock protein 90 alpha family class B member 1
HSPA1A	ENSG00000204389	heat shock protein family A (Hsp70) member 1A
HSPA2	ENSG00000126803	heat shock protein family A (Hsp70) member 2
HSPA4	ENSG00000170606	heat shock protein family A (Hsp70) member 4
HSPA9	ENSG00000113013	heat shock protein family A (Hsp70) member 9
HSPB7	ENSG00000173641	heat shock protein family B (small) member 7
HSPD1	ENSG00000144381	heat shock protein family D (Hsp60) member 1
HSPE1	ENSG00000115541	heat shock protein family E (Hsp10) member 1
HTATIP2	ENSG00000109854	HIV-1 Tat interactive protein 2
HTRA2	ENSG00000115317	HtrA serine peptidase 2
HTT	ENSG00000197386	huntingtin
IARS2	ENSG00000067704	isoleucyl-tRNA synthetase 2 mitochondrial
IBA57	ENSG00000181873	iron-sulfur cluster assembly factor IBA57
ICT1	ENSG00000167862	mitochondrial ribosomal protein L58
IDE	ENSG00000119912	insulin degrading enzyme
IDH1	ENSG00000138413	isocitrate dehydrogenase (NADP(+)) 1
IDH2	ENSG00000182054	isocitrate dehydrogenase (NADP(+)) 2
IDH3A	ENSG00000166411	isocitrate dehydrogenase (NAD(+)) 3 catalytic subunit alpha
IDH3B	ENSG00000101365	isocitrate dehydrogenase (NAD(+)) 3 non-catalytic subunit beta
IDH3G	ENSG00000067829	isocitrate dehydrogenase (NAD(+)) 3 non-catalytic subunit gamma
IDI1	ENSG00000067064	isopentenyl-diphosphate delta isomerase 1
IFI27	ENSG00000165949	interferon alpha inducible protein 27
IFI6	ENSG00000126709	interferon alpha inducible protein 6

IFIT3	ENSG00000119917	interferon induced protein with tetratricopeptide repeats 3
ILF3	ENSG00000129351	interleukin enhancer binding factor 3
IMMP1L	ENSG00000148950	inner mitochondrial membrane peptidase subunit 1
IMMP2L	ENSG00000184903	inner mitochondrial membrane peptidase subunit 2
IMMT	ENSG00000132305	inner membrane mitochondrial protein
IQCE	ENSG00000106012	IQ motif containing E
IREB2	ENSG00000136381	iron responsive element binding protein 2
ISCA1	ENSG00000135070	iron-sulfur cluster assembly 1
ISCA2	ENSG00000165898	iron-sulfur cluster assembly 2
ISCU	ENSG00000136003	iron-sulfur cluster assembly enzyme
ISOC2	ENSG00000063241	isochorismatase domain containing 2
IVD	ENSG00000128928	isovaleryl-CoA dehydrogenase
JTB	ENSG00000143543	jumping translocation breakpoint
KANK2	ENSG00000197256	KN motif and ankyrin repeat domains 2
KARS	ENSG00000065427	lysyl-tRNA synthetase 1
KCNJ11	ENSG00000187486	potassium inwardly rectifying channel subfamily J member 11
KCNJ8	ENSG00000121361	potassium inwardly rectifying channel subfamily J member 8
KIAA0100	ENSG00000007202	bridge-like lipid transfer protein family member 2
KIAA0141	ENSG00000081791	DAP3 binding cell death enhancer 1
KIAA0391	ENSG00000100890	protein only RNase P catalytic subunit
KIAA1279	ENSG00000198954	kinesin family binding protein
KIAA1683	ENSG00000130518	IQ motif containing N
KIF1B	ENSG00000054523	kinesin family member 1B
KLK6	ENSG00000167755	kallikrein related peptidase 6
KMO	ENSG00000117009	kynurenine 3-monooxygenase
KRAS	ENSG00000133703	KRAS proto-oncogene GTPase
KRT5	ENSG00000186081	keratin 5
KYNU	ENSG00000115919	kynureninase
L2HGDH	ENSG00000087299	L-2-hydroxyglutarate dehydrogenase
LACE1	ENSG00000135537	AFG1 like ATPase
LACTB	ENSG00000103642	lactamase beta
LACTB2	ENSG00000147592	lactamase beta 2
LAMC1	ENSG00000135862	laminin subunit gamma 1
LAP3	ENSG00000002549	leucine aminopeptidase 3
LARS2	ENSG00000011376	leucyl-tRNA synthetase 2 mitochondrial
LBR	ENSG00000143815	lamin B receptor
LDHA	ENSG00000134333	lactate dehydrogenase A
LDHAL6B	ENSG00000171989	lactate dehydrogenase A like 6B
LDHB	ENSG00000111716	lactate dehydrogenase B
LDHD	ENSG00000166816	lactate dehydrogenase D
LEPR	ENSG00000116678	leptin receptor
LETM1	ENSG00000168924	leucine zipper and EF-hand containing transmembrane protein 1
LETM2	ENSG00000165046	leucine zipper and EF-hand containing transmembrane protein 2
LETMD1	ENSG00000050426	LETM1 domain containing 1
LIAS	ENSG00000121897	lipoic acid synthetase
LIG1	ENSG00000105486	DNA ligase 1
LIG3	ENSG00000005156	DNA ligase 3
LIPF	ENSG00000182333	lipase F gastric type
LIPT1	ENSG00000144182	lipoyltransferase 1
LIPT2	ENSG00000175536	lipoyl(octanoyl) transferase 2
LONP1	ENSG00000196365	lon peptidase 1 mitochondrial
LONP2	ENSG00000102910	lon peptidase 2 peroxisomal

LRP5	ENSG00000162337	LDL receptor related protein 5
LRPPRC	ENSG00000138095	leucine rich pentatricopeptide repeat containing
LRRC75A-AS1	ENSG00000175061	small nucleolar RNA host gene 29
LRRK1	ENSG00000154237	leucine rich repeat kinase 1
LRRK2	ENSG00000188906	leucine rich repeat kinase 2
LYPLA1	ENSG00000120992	lysophospholipase 1
LYPLAL1	ENSG00000143353	lysophospholipase like 1
LYRM1	ENSG00000102897	LYR motif containing 1
LYRM2	ENSG00000083099	LYR motif containing 2
LYRM4	ENSG00000214113	LYR motif containing 4
LYRM5	ENSG00000205707	electron transfer flavoprotein regulatory factor 1
MAATS1	ENSG00000183833	cilia and flagella associated protein 91
MALSU1	ENSG00000156928	mitochondrial assembly of ribosomal large subunit 1
MAOA	ENSG00000189221	monoamine oxidase A
MAOB	ENSG00000069535	monoamine oxidase B
MAP2K1	ENSG00000169032	mitogen-activated protein kinase kinase 1
MAP2K2	ENSG00000126934	mitogen-activated protein kinase kinase 2
MAPK1	ENSG00000100030	mitogen-activated protein kinase 1
MAPK10	ENSG00000109339	mitogen-activated protein kinase 10
MAPK12	ENSG00000188130	mitogen-activated protein kinase 12
MAPK14	ENSG00000112062	mitogen-activated protein kinase 14
MAPK3	ENSG00000102882	mitogen-activated protein kinase 3
MAPK8	ENSG00000107643	mitogen-activated protein kinase 8
MAPK8IP1	ENSG00000121653	mitogen-activated protein kinase 8 interacting protein 1
MAPK9	ENSG00000050748	mitogen-activated protein kinase 9
MARC1	ENSG00000186205	mitochondrial amidoxime reducing component 1
MARC2	ENSG00000117791	mitochondrial amidoxime reducing component 2
MARCH5	ENSG00000198060	membrane associated ring-CH-type finger 5
MARK2	ENSG00000072518	microtubule affinity regulating kinase 2
MARS	ENSG00000166986	methionyl-tRNA synthetase 1
MAT2B	ENSG00000038274	methionine adenosyltransferase 2B
MAVS	ENSG00000088888	mitochondrial antiviral signaling protein
MCAT	ENSG00000100294	malonyl-CoA-acyl carrier protein transacylase
MCCC1	ENSG00000078070	methylcrotonyl-CoA carboxylase subunit 1
MCCC2	ENSG00000131844	methylcrotonyl-CoA carboxylase subunit 2
MCCD1	ENSG00000204511	mitochondrial coiled-coil domain 1
MCEE	ENSG00000124370	methylmalonyl-CoA epimerase
MCL1	ENSG00000143384	MCL1 apoptosis regulator BCL2 family member
MCUR1	ENSG00000050393	mitochondrial calcium uniporter regulator 1
MDH1	ENSG00000014641	malate dehydrogenase 1
MDH2	ENSG00000146701	malate dehydrogenase 2
ME1	ENSG00000065833	malic enzyme 1
ME2	ENSG00000082212	malic enzyme 2
ME3	ENSG00000151376	malic enzyme 3
MECR	ENSG00000116353	mitochondrial trans-2-enoyl-CoA reductase
MED7	ENSG00000155868	mediator complex subunit 7
METAP1D	ENSG00000172878	methionyl aminopeptidase type 1D mitochondrial
METTL12	ENSG00000214756	citrate synthase lysine methyltransferase
METTL15	ENSG00000169519	methyltransferase like 15
METTL17	ENSG00000165792	methyltransferase like 17
METTL5	ENSG00000138382	methyltransferase 5 N6-adenosine
METTL8	ENSG00000123600	methyltransferase 8 methylcytidine

MFF	ENSG00000168958	mitochondrial fission factor
MFN1	ENSG00000171109	mitofusin 1
MGME1	ENSG00000125871	mitochondrial genome maintenance exonuclease 1
MGST1	ENSG00000008394	microsomal glutathione S-transferase 1
MGST3	ENSG00000143198	microsomal glutathione S-transferase 3
MICOS13	ENSG00000174917	mitochondrial contact site and cristae organizing system subunit 13
MIEF2	ENSG00000177427	mitochondrial elongation factor 2
MINOS1-NBL1	ENSG00000270136	MICOS10-NBL1 readthrough
MIPEP	ENSG00000027001	mitochondrial intermediate peptidase
MLH1	ENSG00000076242	mutL homolog 1
MLYCD	ENSG00000103150	malonyl-CoA decarboxylase
MMAB	ENSG00000139428	metabolism of cobalamin associated B
MMACHC	ENSG00000132763	metabolism of cobalamin associated C
MMADHC	ENSG00000168288	metabolism of cobalamin associated D
MMP2	ENSG00000087245	matrix metallopeptidase 2
MOAP1	ENSG00000165943	modulator of apoptosis 1
MOBP	ENSG00000168314	myelin associated oligodendrocyte basic protein
MOCS1	ENSG00000124615	molybdenum cofactor synthesis 1
MPC2	ENSG00000143158	mitochondrial pyruvate carrier 2
MPDU1	ENSG00000129255	mannose-P-dolichol utilization defect 1
MPO	ENSG00000005381	myeloperoxidase
MPP7	ENSG00000150054	MAGUK p55 scaffold protein 7
MPST	ENSG00000128309	mercaptopyruvate sulfurtransferase
MPV17	ENSG00000115204	mitochondrial inner membrane protein MPV17
MPV17L	ENSG00000156968	MPV17 mitochondrial inner membrane protein like
MPV17L2	ENSG00000254858	MPV17 mitochondrial inner membrane protein like 2
MRM1	ENSG00000278619	mitochondrial rRNA methyltransferase 1
MRPL1	ENSG00000169288	mitochondrial ribosomal protein L1
MRPL10	ENSG00000159111	mitochondrial ribosomal protein L10
MRPL11	ENSG00000174547	mitochondrial ribosomal protein L11
MRPL12	ENSG00000262814	mitochondrial ribosomal protein L12
MRPL13	ENSG00000172172	mitochondrial ribosomal protein L13
MRPL14	ENSG00000180992	mitochondrial ribosomal protein L14
MRPL15	ENSG00000137547	mitochondrial ribosomal protein L15
MRPL16	ENSG00000166902	mitochondrial ribosomal protein L16
MRPL17	ENSG00000158042	mitochondrial ribosomal protein L17
MRPL18	ENSG00000112110	mitochondrial ribosomal protein L18
MRPL19	ENSG00000115364	mitochondrial ribosomal protein L19
MRPL2	ENSG00000112651	mitochondrial ribosomal protein L2
MRPL20	ENSG00000242485	mitochondrial ribosomal protein L20
MRPL21	ENSG00000197345	mitochondrial ribosomal protein L21
MRPL22	ENSG00000082515	mitochondrial ribosomal protein L22
MRPL23	ENSG00000214026	mitochondrial ribosomal protein L23
MRPL24	ENSG00000143314	mitochondrial ribosomal protein L24
MRPL27	ENSG00000108826	mitochondrial ribosomal protein L27
MRPL28	ENSG00000086504	mitochondrial ribosomal protein L28
MRPL3	ENSG00000114686	mitochondrial ribosomal protein L3
MRPL30	ENSG00000185414	mitochondrial ribosomal protein L30
MRPL32	ENSG00000106591	mitochondrial ribosomal protein L32
MRPL33	ENSG00000243147	mitochondrial ribosomal protein L33
MRPL34	ENSG00000130312	mitochondrial ribosomal protein L34
MRPL35	ENSG00000132313	mitochondrial ribosomal protein L35

MRPL36	ENSG00000171421	mitochondrial ribosomal protein L36
MRPL37	ENSG00000116221	mitochondrial ribosomal protein L37
MRPL38	ENSG00000204316	mitochondrial ribosomal protein L38
MRPL39	ENSG00000154719	mitochondrial ribosomal protein L39
MRPL4	ENSG00000105364	mitochondrial ribosomal protein L4
MRPL40	ENSG00000185608	mitochondrial ribosomal protein L40
MRPL41	ENSG00000182154	mitochondrial ribosomal protein L41
MRPL42	ENSG00000198015	mitochondrial ribosomal protein L42
MRPL43	ENSG00000055950	mitochondrial ribosomal protein L43
MRPL44	ENSG00000135900	mitochondrial ribosomal protein L44
MRPL45	ENSG00000278845	mitochondrial ribosomal protein L45
MRPL46	ENSG00000259494	mitochondrial ribosomal protein L46
MRPL47	ENSG00000136522	mitochondrial ribosomal protein L47
MRPL48	ENSG00000175581	mitochondrial ribosomal protein L48
MRPL49	ENSG00000149792	mitochondrial ribosomal protein L49
MRPL50	ENSG00000136897	mitochondrial ribosomal protein L50
MRPL51	ENSG00000111639	mitochondrial ribosomal protein L51
MRPL52	ENSG00000172590	mitochondrial ribosomal protein L52
MRPL53	ENSG00000204822	mitochondrial ribosomal protein L53
MRPL54	ENSG00000183617	mitochondrial ribosomal protein L54
MRPL57	ENSG00000173141	mitochondrial ribosomal protein L57
MRPL9	ENSG00000143436	mitochondrial ribosomal protein L9
MRPS10	ENSG00000048544	mitochondrial ribosomal protein S10
MRPS11	ENSG00000181991	mitochondrial ribosomal protein S11
MRPS12	ENSG00000128626	mitochondrial ribosomal protein S12
MRPS14	ENSG00000120333	mitochondrial ribosomal protein S14
MRPS15	ENSG00000116898	mitochondrial ribosomal protein S15
MRPS16	ENSG00000182180	mitochondrial ribosomal protein S16
MRPS17	ENSG00000239789	mitochondrial ribosomal protein S17
MRPS18A	ENSG00000096080	mitochondrial ribosomal protein S18A
MRPS18B	ENSG00000204568	mitochondrial ribosomal protein S18B
MRPS18C	ENSG00000163319	mitochondrial ribosomal protein S18C
MRPS2	ENSG00000122140	mitochondrial ribosomal protein S2
MRPS21	ENSG00000266472	mitochondrial ribosomal protein S21
MRPS22	ENSG00000175110	mitochondrial ribosomal protein S22
MRPS23	ENSG00000181610	mitochondrial ribosomal protein S23
MRPS24	ENSG00000062582	mitochondrial ribosomal protein S24
MRPS25	ENSG00000131368	mitochondrial ribosomal protein S25
MRPS26	ENSG00000125901	mitochondrial ribosomal protein S26
MRPS27	ENSG00000113048	mitochondrial ribosomal protein S27
MRPS28	ENSG00000147586	mitochondrial ribosomal protein S28
MRPS30	ENSG00000112996	mitochondrial ribosomal protein S30
MRPS31	ENSG00000102738	mitochondrial ribosomal protein S31
MRPS33	ENSG00000090263	mitochondrial ribosomal protein S33
MRPS34	ENSG00000074071	mitochondrial ribosomal protein S34
MRPS35	ENSG00000061794	mitochondrial ribosomal protein S35
MRPS36	ENSG00000134056	mitochondrial ribosomal protein S36
MRPS5	ENSG00000144029	mitochondrial ribosomal protein S5
MRPS6	ENSG00000243927	mitochondrial ribosomal protein S6
MRPS7	ENSG00000125445	mitochondrial ribosomal protein S7
MRPS9	ENSG00000135972	mitochondrial ribosomal protein S9
MRRF	ENSG00000148187	mitochondrial ribosome recycling factor

MRS2	ENSG00000124532	magnesium transporter MRS2
MSRA	ENSG00000175806	methionine sulfoxide reductase A
MSRB2	ENSG00000148450	methionine sulfoxide reductase B2
MSRB3	ENSG00000174099	methionine sulfoxide reductase B3
MT-ATP6	ENSG00000198899	mitochondrially encoded ATP synthase membrane subunit 6
MT-ATP8	ENSG00000228253	mitochondrially encoded ATP synthase membrane subunit 8
MT-CO1	ENSG00000198804	mitochondrially encoded cytochrome c oxidase I
MT-CO2	ENSG00000198712	mitochondrially encoded cytochrome c oxidase II
MT-CO3	ENSG00000198938	mitochondrially encoded cytochrome c oxidase III
MT-CYB	ENSG00000198727	mitochondrially encoded cytochrome b
MT-CYB	ENSG00000198727	mitochondrially encoded cytochrome b
MT-ND1	ENSG00000198888	mitochondrially encoded NADH:ubiquinone oxidoreductase core subunit 1
MT-ND2	ENSG00000198763	mitochondrially encoded NADH:ubiquinone oxidoreductase core subunit 2
MT-ND3	ENSG00000198840	mitochondrially encoded NADH:ubiquinone oxidoreductase core subunit 3
MT-ND4	ENSG00000198886	mitochondrially encoded NADH:ubiquinone oxidoreductase core subunit 4
MT-ND4L	ENSG00000212907	mitochondrially encoded NADH:ubiquinone oxidoreductase core subunit 4L
MT-ND5	ENSG00000198786	mitochondrially encoded NADH:ubiquinone oxidoreductase core subunit 5
MT-ND6	ENSG00000198695	mitochondrially encoded NADH:ubiquinone oxidoreductase core subunit 6
MT-RNR2	ENSG00000210082	mitochondrially encoded 16S rRNA
MTCH1	ENSG00000137409	mitochondrial carrier 1
MTERF4	ENSG00000122085	mitochondrial transcription termination factor 4
MTERFD1	ENSG00000156469	mitochondrial transcription termination factor 3
MTERFD2	ENSG00000122085	mitochondrial transcription termination factor 4
MTERFD3	ENSG00000120832	mitochondrial transcription termination factor 2
MTFMT	ENSG00000103707	mitochondrial methionyl-tRNA formyltransferase
MTFP1	ENSG00000242114	mitochondrial fission process 1
MTFR2	ENSG00000146410	mitochondrial fission regulator 2
MTG2	ENSG00000101181	mitochondrial ribosome associated GTPase 2
MTHFD1	ENSG00000100714	methylenetetrahydrofolate dehydrogenase cyclohydrolase and formyltetrahydrofolate synthetase 1
MTHFD1L	ENSG00000120254	methylenetetrahydrofolate dehydrogenase (NADP+ dependent) 1 like
MTHFD2	ENSG00000065911	methylenetetrahydrofolate dehydrogenase (NADP+ dependent) 2 methenyltetrahydrofolate cyclohydrolase
MTHFD2L	ENSG00000163738	methylenetetrahydrofolate dehydrogenase (NADP+ dependent) 2 like
MTHFS	ENSG00000136371	methenyltetrahydrofolate synthetase
MTIF2	ENSG00000085760	mitochondrial translational initiation factor 2
MTIF3	ENSG00000122033	mitochondrial translational initiation factor 3
MTO1	ENSG00000135297	mitochondrial tRNA translation optimization 1
MTPAP	ENSG00000107951	mitochondrial poly(A) polymerase
MTRF1	ENSG00000120662	mitochondrial translation release factor 1
MTUS1	ENSG00000129422	microtubule associated scaffold protein 1
MTX1	ENSG00000173171	metaxin 1
MTX2	ENSG00000128654	metaxin 2
MUL1	ENSG00000090432	mitochondrial E3 ubiquitin protein ligase 1
MUT	ENSG00000146085	methylmalonyl-CoA mutase

MUTYH	ENSG00000132781	mutY DNA glycosylase
MYCBP	ENSG00000214114	MYC binding protein
MYL10	ENSG00000106436	myosin light chain 10
MYO1C	ENSG00000197879	myosin IC
NADK2	ENSG00000152620	NAD kinase 2 mitochondrial
NAGS	ENSG00000161653	N-acetylglutamate synthase
NAIF1	ENSG00000171169	nuclear apoptosis inducing factor 1
NAPG	ENSG00000134265	NSF attachment protein gamma
NARS	ENSG00000134440	asparaginyl-tRNA synthetase 1
NARS2	ENSG00000137513	asparaginyl-tRNA synthetase 2 mitochondrial
NBR1	ENSG00000188554	NBR1 autophagy cargo receptor
NCBP1	ENSG00000136937	nuclear cap binding protein subunit 1
NCEH1	ENSG00000144959	neutral cholesterol ester hydrolase 1
NCOA4	ENSG00000266412	nuclear receptor coactivator 4
NDFIP2	ENSG00000102471	Nedd4 family interacting protein 2
NDUFA1	ENSG00000125356	NADH:ubiquinone oxidoreductase subunit A1
NDUFA10	ENSG00000130414	NADH:ubiquinone oxidoreductase subunit A10
NDUFA11	ENSG00000174886	NADH:ubiquinone oxidoreductase subunit A11
NDUFA12	ENSG00000184752	NADH:ubiquinone oxidoreductase subunit A12
NDUFA13	ENSG00000186010	NADH:ubiquinone oxidoreductase subunit A13
NDUFA2	ENSG00000131495	NADH:ubiquinone oxidoreductase subunit A2
NDUFA3	ENSG00000170906	NADH:ubiquinone oxidoreductase subunit A3
NDUFA4	ENSG00000189043	NDUFA4 mitochondrial complex associated
NDUFA5	ENSG00000128609	NADH:ubiquinone oxidoreductase subunit A5
NDUFA6	ENSG00000184983	NADH:ubiquinone oxidoreductase subunit A6
NDUFA7	ENSG00000267855	NADH:ubiquinone oxidoreductase subunit A7
NDUFA8	ENSG00000119421	NADH:ubiquinone oxidoreductase subunit A8
NDUFA9	ENSG00000139180	NADH:ubiquinone oxidoreductase subunit A9
NDUFAB1	ENSG00000004779	NADH:ubiquinone oxidoreductase subunit AB1
NDUFAF1	ENSG00000137806	NADH:ubiquinone oxidoreductase complex assembly factor 1
NDUFAF2	ENSG00000164182	NADH:ubiquinone oxidoreductase complex assembly factor 2
NDUFAF3	ENSG00000178057	NADH:ubiquinone oxidoreductase complex assembly factor 3
NDUFAF7	ENSG00000003509	NADH:ubiquinone oxidoreductase complex assembly factor 7
NDUFB1	ENSG00000183648	NADH:ubiquinone oxidoreductase subunit B1
NDUFB10	ENSG00000140990	NADH:ubiquinone oxidoreductase subunit B10
NDUFB11	ENSG00000147123	NADH:ubiquinone oxidoreductase subunit B11
NDUFB2	ENSG00000090266	NADH:ubiquinone oxidoreductase subunit B2
NDUFB3	ENSG00000119013	NADH:ubiquinone oxidoreductase subunit B3
NDUFB4	ENSG00000065518	NADH:ubiquinone oxidoreductase subunit B4
NDUFB5	ENSG00000136521	NADH:ubiquinone oxidoreductase subunit B5
NDUFB6	ENSG00000165264	NADH:ubiquinone oxidoreductase subunit B6
NDUFB7	ENSG00000099795	NADH:ubiquinone oxidoreductase subunit B7
NDUFB8	ENSG00000166136	NADH:ubiquinone oxidoreductase subunit B8
NDUFB9	ENSG00000147684	NADH:ubiquinone oxidoreductase subunit B9
NDUFC1	ENSG00000109390	NADH:ubiquinone oxidoreductase subunit C1
NDUFC2	ENSG00000151366	NADH:ubiquinone oxidoreductase subunit C2
NDUFS1	ENSG00000023228	NADH:ubiquinone oxidoreductase core subunit S1
NDUFS2	ENSG00000158864	NADH:ubiquinone oxidoreductase core subunit S2
NDUFS3	ENSG00000213619	NADH:ubiquinone oxidoreductase core subunit S3
NDUFS4	ENSG00000164258	NADH:ubiquinone oxidoreductase subunit S4
NDUFS5	ENSG00000168653	NADH:ubiquinone oxidoreductase subunit S5
NDUFS6	ENSG00000145494	NADH:ubiquinone oxidoreductase subunit S6

NDUFS7	ENSG00000115286	NADH:ubiquinone oxidoreductase core subunit S7
NDUFS8	ENSG00000110717	NADH:ubiquinone oxidoreductase core subunit S8
NDUFV1	ENSG00000167792	NADH:ubiquinone oxidoreductase core subunit V1
NDUFV2	ENSG00000178127	NADH:ubiquinone oxidoreductase core subunit V2
NDUFV3	ENSG00000160194	NADH:ubiquinone oxidoreductase subunit V3
NEFH	ENSG00000100285	neurofilament heavy chain
NEU4	ENSG00000204099	neuraminidase 4
NFKB1	ENSG00000109320	nuclear factor kappa B subunit 1
NFS1	ENSG00000244005	NFS1 cysteine desulfurase
NFU1	ENSG00000169599	NFU1 iron-sulfur cluster scaffold
NGB	ENSG00000165553	neuroglobin
NGRN	ENSG00000182768	neugrin neurite outgrowth associated
NIF3L1	ENSG00000196290	NGG1 interacting factor 3 like 1
NIPSNAP1	ENSG00000184117	nipsnap homolog 1
NIPSNAP3A	ENSG00000136783	nipsnap homolog 3A
NIPSNAP3B	ENSG00000165028	nipsnap homolog 3B
NIT1	ENSG00000158793	nitrilase 1
NIT2	ENSG00000114021	nitrilase family member 2
NLN	ENSG00000123213	neurolysin
NLRP5	ENSG00000171487	NLR family pyrin domain containing 5
NLRX1	ENSG00000160703	NLR family member X1
NME1	ENSG00000239672	NME/NM23 nucleoside diphosphate kinase 1
NME3	ENSG00000103024	NME/NM23 nucleoside diphosphate kinase 3
NME4	ENSG00000103202	NME/NM23 nucleoside diphosphate kinase 4
NMNAT3	ENSG00000163864	nicotinamide nucleotide adenylyltransferase 3
NNT	ENSG00000112992	nicotinamide nucleotide transhydrogenase
NOA1	ENSG00000084092	nitric oxide associated 1
NOL3	ENSG00000140939	nucleolar protein 3
NOL6	ENSG00000165271	nucleolar protein 6
NOP14	ENSG00000087269	NOP14 nucleolar protein
NOS1	ENSG00000089250	nitric oxide synthase 1
NOX4	ENSG00000086991	NADPH oxidase 4
NPTX1	ENSG00000171246	neuronal pentraxin 1
NRD1	ENSG00000078618	nardilysin convertase
NSUN3	ENSG00000178694	NOP2/Sun RNA methyltransferase 3
NSUN4	ENSG00000117481	NOP2/Sun RNA methyltransferase 4
NT5C3A	ENSG00000122643	5'-nucleotidase cytosolic IIIA
NT5DC2	ENSG00000168268	5'-nucleotidase domain containing 2
NT5DC3	ENSG00000111696	5'-nucleotidase domain containing 3
NT5M	ENSG00000205309	5' 3'-nucleotidase mitochondrial
NTHL1	ENSG00000065057	nth like DNA glycosylase 1
NUBPL	ENSG00000151413	NUBP iron-sulfur cluster assembly factor mitochondrial
NUCB2	ENSG00000070081	nucleobindin 2
NUDT1	ENSG00000106268	nudix hydrolase 1
NUDT13	ENSG00000166321	nudix hydrolase 13
NUDT19	ENSG00000213965	nudix hydrolase 19
NUDT2	ENSG00000164978	nudix hydrolase 2
NUDT5	ENSG00000165609	nudix hydrolase 5
NUDT6	ENSG00000170917	nudix hydrolase 6
NUDT8	ENSG00000167799	nudix hydrolase 8
NUDT9	ENSG00000170502	nudix hydrolase 9
NXNL1	ENSG00000171773	nucleoredoxin like 1

OAS1	ENSG00000089127	2'-5'-oligoadenylate synthetase 1
OAS2	ENSG00000111335	2'-5'-oligoadenylate synthetase 2
OAT	ENSG00000065154	ornithine aminotransferase
OBSCN	ENSG00000154358	obscurin cytoskeletal calmodulin and titin-interacting RhoGEF
OCIAD1	ENSG00000109180	OCIA domain containing 1
OCIAD2	ENSG00000145247	OCIA domain containing 2
OGDH	ENSG00000105953	oxoglutarate dehydrogenase
OGDHL	ENSG00000197444	oxoglutarate dehydrogenase L
OGG1	ENSG00000114026	8-oxoguanine DNA glycosylase
OGT	ENSG00000147162	O-linked N-acetylglucosamine (GlcNAc) transferase
OLFM4	ENSG00000102837	olfactomedin 4
OMA1	ENSG00000162600	OMA1 zinc metallopeptidase
OPA1	ENSG00000198836	OPA1 mitochondrial dynamin like GTPase
OPA3	ENSG00000125741	outer mitochondrial membrane lipid metabolism regulator OPA3
OPN1SW	ENSG00000128617	opsin 1 short wave sensitive
OSBPL1A	ENSG00000141447	oxysterol binding protein like 1A
OSGEPL1	ENSG00000128694	O-sialoglycoprotein endopeptidase like 1
OTC	ENSG00000036473	ornithine transcarbamylase
OXA1L	ENSG00000155463	OXA1L mitochondrial inner membrane protein
OXCT1	ENSG00000083720	3-oxoacid CoA-transferase 1
OXLD1	ENSG00000204237	oxidoreductase like domain containing 1
OXNAD1	ENSG00000154814	oxidoreductase NAD binding domain containing 1
OXR1	ENSG00000164830	oxidation resistance 1
OXSM	ENSG00000151093	3-oxoacyl-ACP synthase mitochondrial
P4HA1	ENSG00000122884	prolyl 4-hydroxylase subunit alpha 1
P4HB	ENSG00000185624	prolyl 4-hydroxylase subunit beta
PABPC5	ENSG00000174740	poly(A) binding protein cytoplasmic 5
PACRG	ENSG00000112530	parkin coregulated
PACS2	ENSG00000179364	phosphofurin acidic cluster sorting protein 2
PACSIN2	ENSG00000100266	protein kinase C and casein kinase substrate in neurons 2
PAICS	ENSG00000128050	phosphoribosylaminoimidazole carboxylase and phosphoribosylaminoimidazolesuccinocarboxamide synthase
PAK7	ENSG00000101349	p21 (RAC1) activated kinase 5
PAM16	ENSG00000217930	presequence translocase associated motor 16
PANK2	ENSG00000125779	pantothenate kinase 2
PARG	ENSG00000227345	poly(ADP-ribose) glycohydrolase
PARK2	ENSG00000185345	parkin RBR E3 ubiquitin protein ligase
PARK7	ENSG00000116288	Parkinsonism associated deglycase
PARL	ENSG00000175193	presenilin associated rhomboid like
PARS2	ENSG00000162396	prolyl-tRNA synthetase 2 mitochondrial
PC	ENSG00000173599	pyruvate carboxylase
PCBD2	ENSG00000132570	pterin-4 alpha-carbinolamine dehydratase 2
PCCA	ENSG00000175198	propionyl-CoA carboxylase subunit alpha
PCCB	ENSG00000114054	propionyl-CoA carboxylase subunit beta
PCK2	ENSG00000100889	phosphoenolpyruvate carboxykinase 2 mitochondrial
PDF	ENSG00000258429	peptide deformylase mitochondrial
PDHA1	ENSG00000131828	pyruvate dehydrogenase E1 subunit alpha 1
PDHA2	ENSG00000163114	pyruvate dehydrogenase E1 subunit alpha 2
PDHB	ENSG00000168291	pyruvate dehydrogenase E1 subunit beta
PDHX	ENSG00000110435	pyruvate dehydrogenase complex component X
PDK1	ENSG00000152256	pyruvate dehydrogenase kinase 1
PDK2	ENSG00000005882	pyruvate dehydrogenase kinase 2

PDK3	ENSG00000067992	pyruvate dehydrogenase kinase 3
PDK4	ENSG00000004799	pyruvate dehydrogenase kinase 4
PDP1	ENSG00000164951	pyruvate dehydrogenase phosphatase catalytic subunit 1
PDP2	ENSG00000172840	pyruvate dehydrogenase phosphatase catalytic subunit 2
PDPR	ENSG00000090857	pyruvate dehydrogenase phosphatase regulatory subunit
PDSS1	ENSG00000148459	decaprenyl diphosphate synthase subunit 1
PDSS2	ENSG00000164494	decaprenyl diphosphate synthase subunit 2
PECR	ENSG00000115425	peroxisomal trans-2-enoyl-CoA reductase
PEMT	ENSG00000133027	phosphatidylethanolamine N-methyltransferase
PET100	ENSG00000229833	PET100 cytochrome c oxidase chaperone
PET117	ENSG00000232838	PET117 cytochrome c oxidase chaperone
PEX11B	ENSG00000131779	peroxisomal biogenesis factor 11 beta
PEX5	ENSG00000139197	peroxisomal biogenesis factor 5
PFDN2	ENSG00000143256	prefoldin subunit 2
PFDN4	ENSG00000101132	prefoldin subunit 4
PGAM5	ENSG00000247077	PGAM family member 5 mitochondrial serine/threonine protein phosphatase
PGS1	ENSG00000087157	phosphatidylglycerophosphate synthase 1
PHB	ENSG00000167085	prohibitin 1
PHB2	ENSG00000215021	prohibitin 2
PHYH	ENSG00000107537	phytanoyl-CoA 2-hydroxylase
PHYKPL	ENSG00000175309	5-phosphohydroxy-L-lysine phospho-lyase
PI4K2A	ENSG00000155252	phosphatidylinositol 4-kinase type 2 alpha
PI4KA	ENSG00000241973	phosphatidylinositol 4-kinase alpha
PICK1	ENSG00000100151	protein interacting with PRKCA 1
PIF1	ENSG00000140451	PIF1 5'-to-3' DNA helicase
PIN4	ENSG00000102309	peptidylprolyl cis/trans isomerase NIMA-interacting 4
PINK1	ENSG00000158828	PTEN induced kinase 1
PISD	ENSG00000241878	phosphatidylserine decarboxylase
PITRM1	ENSG00000107959	pitrilysin metallopeptidase 1
PKM	ENSG00000067225	pyruvate kinase M1/2
PLA2G15	ENSG00000103066	phospholipase A2 group XV
PLA2G2A	ENSG00000188257	phospholipase A2 group IIA
PLGRKT	ENSG00000107020	plasminogen receptor with a C-terminal lysine
PLIN5	ENSG00000214456	perilipin 5
PLN	ENSG00000198523	phospholamban
PLSCR3	ENSG00000187838	phospholipid scramblase 3
PMAIP1	ENSG00000141682	phorbol-12-myristate-13-acetate-induced protein 1
PMPCA	ENSG00000165688	peptidase mitochondrial processing subunit alpha
PMPCB	ENSG00000105819	peptidase mitochondrial processing subunit beta
PNKD	ENSG00000127838	PNKD metallo-beta-lactamase domain containing
PNPLA7	ENSG00000130653	patatin like phospholipase domain containing 7
PNPLA8	ENSG00000135241	patatin like phospholipase domain containing 8
PNPO	ENSG00000108439	pyridoxamine 5'-phosphate oxidase
PNPT1	ENSG00000138035	polyribonucleotide nucleotidyltransferase 1
POLD3	ENSG00000077514	DNA polymerase delta 3 accessory subunit
POLDIP2	ENSG00000004142	DNA polymerase delta interacting protein 2
POLG	ENSG00000140521	DNA polymerase gamma catalytic subunit
POLG2	ENSG00000256525	DNA polymerase gamma 2 accessory subunit
POLRMT	ENSG00000099821	RNA polymerase mitochondrial
PON2	ENSG00000105854	paraoxonase 2
POR	ENSG00000127948	cytochrome p450 oxidoreductase

PPA2	ENSG00000138777	inorganic pyrophosphatase 2
PPARGC1B	ENSG00000155846	PPARG coactivator 1 beta
PPIF	ENSG00000108179	peptidylprolyl isomerase F
PPL	ENSG00000118898	periplakin
PPM1E	ENSG00000175175	protein phosphatase Mg2+/Mn2+ dependent 1E
PPM1K	ENSG00000163644	protein phosphatase Mg2+/Mn2+ dependent 1K
PPOX	ENSG00000143224	protoporphyrinogen oxidase
PPP1CC	ENSG00000186298	protein phosphatase 1 catalytic subunit gamma
PPP2CA	ENSG00000113575	protein phosphatase 2 catalytic subunit alpha
PPP2R1A	ENSG00000105568	protein phosphatase 2 scaffold subunit Aalpha
PPP2R2B	ENSG00000156475	protein phosphatase 2 regulatory subunit Bbeta
PPP3CA	ENSG00000138814	protein phosphatase 3 catalytic subunit alpha
PPTC7	ENSG00000196850	protein phosphatase targeting COQ7
PPWD1	ENSG00000113593	peptidylprolyl isomerase domain and WD repeat containing 1
PRDX2	ENSG00000167815	peroxiredoxin 2
PRDX3	ENSG00000165672	peroxiredoxin 3
PRDX4	ENSG00000123131	peroxiredoxin 4
PRDX5	ENSG00000126432	peroxiredoxin 5
PRDX6	ENSG00000117592	peroxiredoxin 6
PRELID1	ENSG00000169230	PRELI domain containing 1
PRELID2	ENSG00000186314	PRELI domain containing 2
PREPL	ENSG00000138078	prolyl endopeptidase like
PRKACA	ENSG00000072062	protein kinase cAMP-activated catalytic subunit alpha
PRKCA	ENSG00000154229	protein kinase C alpha
PRKCD	ENSG00000163932	protein kinase C delta
PRKCE	ENSG00000171132	protein kinase C epsilon
PRODH	ENSG00000100033	proline dehydrogenase 1
PRODH2	ENSG00000250799	proline dehydrogenase 2
PROSC	ENSG00000147471	pyridoxal phosphate binding protein
PRR5L	ENSG00000135362	proline rich 5 like
PRSS35	ENSG00000146250	serine protease 35
PSAP	ENSG00000197746	prosaposin
PSEN1	ENSG00000080815	presenilin 1
PSMA6	ENSG00000100902	proteasome 20S subunit alpha 6
PSMB3	ENSG00000277791	proteasome 20S subunit beta 3
PSTK	ENSG00000179988	phosphoseryl-tRNA kinase
PTCD1	ENSG00000106246	pentatricopeptide repeat domain 1
PTCD2	ENSG00000049883	pentatricopeptide repeat domain 2
PTCD3	ENSG00000132300	pentatricopeptide repeat domain 3
PTEN	ENSG00000171862	phosphatase and tensin homolog
PTGES2	ENSG00000148334	prostaglandin E synthase 2
PTPMT1	ENSG00000110536	protein tyrosine phosphatase mitochondrial 1
PTPN11	ENSG00000179295	protein tyrosine phosphatase non-receptor type 11
PTPN4	ENSG00000088179	protein tyrosine phosphatase non-receptor type 4
PTRF	ENSG00000177469	caveolae associated protein 1
PTRH1	ENSG00000187024	peptidyl-tRNA hydrolase 1 homolog
PTRH2	ENSG00000141378	peptidyl-tRNA hydrolase 2
PTS	ENSG00000150787	6-pyruvoyltetrahydropterin synthase
PUS1	ENSG00000177192	pseudouridine synthase 1
PUSL1	ENSG00000169972	pseudouridine synthase like 1
PXMP2	ENSG00000176894	peroxisomal membrane protein 2
PXMP4	ENSG00000101417	peroxisomal membrane protein 4

PYCARD	ENSG00000103490	PYD and CARD domain containing
PYCR1	ENSG00000183010	pyrroline-5-carboxylate reductase 1
Q6ZSR3		
QRSL1	ENSG00000130348	glutaminyl-tRNA amidotransferase subunit QRSL1
QTRT1	ENSG00000213339	queuine tRNA-ribosyltransferase catalytic subunit 1
QTRTD1	ENSG00000151576	queuine tRNA-ribosyltransferase accessory subunit 2
RAB11A	ENSG00000103769	RAB11A member RAS oncogene family
RAB11B	ENSG00000185236	RAB11B member RAS oncogene family
RAB11FIP5	ENSG00000135631	RAB11 family interacting protein 5
RAB1B	ENSG00000174903	RAB1B member RAS oncogene family
RAB24	ENSG00000169228	RAB24 member RAS oncogene family
RAB32	ENSG00000118508	RAB32 member RAS oncogene family
RAB35	ENSG00000111737	RAB35 member RAS oncogene family
RAB3D	ENSG00000105514	RAB3D member RAS oncogene family
RAB40AL	ENSG00000102128	RAB40A like
RAB8B	ENSG00000166128	RAB8B member RAS oncogene family
RAD51	ENSG00000051180	RAD51 recombinase
RAD51C	ENSG00000108384	RAD51 paralog C
RAI14	ENSG00000039560	retinoic acid induced 14
RANBP2	ENSG00000153201	RAN binding protein 2
RAP1GDS1	ENSG00000138698	Rap1 GTPase-GDP dissociation stimulator 1
RARS	ENSG00000113643	arginyl-tRNA synthetase 1
RARS2	ENSG00000146282	arginyl-tRNA synthetase 2 mitochondrial
RBFA	ENSG00000101546	ribosome binding factor A
RCC1L	ENSG00000274523	RCC1 like
RCN2	ENSG00000117906	reticulocalbin 2
RDH11	ENSG00000072042	retinol dehydrogenase 11
RDH13	ENSG00000160439	retinol dehydrogenase 13
RDH14	ENSG00000240857	retinol dehydrogenase 14
RECQL4	ENSG00000160957	RecQ like helicase 4
REEP1	ENSG00000068615	receptor accessory protein 1
REXO2	ENSG00000076043	RNA exonuclease 2
RFK	ENSG00000135002	riboflavin kinase
RGS2	ENSG00000116741	regulator of G protein signaling 2
RHBDD1	ENSG00000144468	rhomboid domain containing 1
RHOA	ENSG00000067560	ras homolog family member A
RHOT1	ENSG00000126858	ras homolog family member T1
RHOT2	ENSG00000140983	ras homolog family member T2
RILP	ENSG00000167705	Rab interacting lysosomal protein
RIPK1	ENSG00000137275	receptor interacting serine/threonine kinase 1
RMDN3	ENSG00000137824	regulator of microtubule dynamics 3
RMND1	ENSG00000155906	required for meiotic nuclear division 1 homolog
RNASEH1	ENSG00000171865	ribonuclease H1
RNASEL	ENSG00000135828	ribonuclease L
RNF168	ENSG00000163961	ring finger protein 168
RNF5	ENSG00000204308	ring finger protein 5
RNMTL1	ENSG00000171861	mitochondrial rRNA methyltransferase 3
ROMO1	ENSG00000125995	reactive oxygen species modulator 1
RPIA	ENSG00000153574	ribose 5-phosphate isomerase A
RPL10A	ENSG00000198755	ribosomal protein L10a
RPL34	ENSG00000109475	ribosomal protein L34
RPL35A	ENSG00000182899	ribosomal protein L35a

RPP14	ENSG00000163684	ribonuclease P/MRP subunit p14
RPS14	ENSG00000164587	ribosomal protein S14
RPS15A	ENSG00000134419	ribosomal protein S15a
RPS18	ENSG00000231500	ribosomal protein S18
RPS6KB1	ENSG00000108443	ribosomal protein S6 kinase B1
RPUSD3	ENSG00000156990	RNA pseudouridine synthase D3
RPUSD4	ENSG00000165526	RNA pseudouridine synthase D4
RRP15	ENSG00000067533	ribosomal RNA processing 15 homolog
RSAD1	ENSG00000136444	radical S-adenosyl methionine domain containing 1
RSAD2	ENSG00000134321	radical S-adenosyl methionine domain containing 2
RTN4IP1	ENSG00000130347	reticulon 4 interacting protein 1
SACS	ENSG00000151835	sacsin molecular chaperone
SAMM50	ENSG00000100347	SAMM50 sorting and assembly machinery component
SARDH	ENSG00000123453	sarcosine dehydrogenase
SARM1	ENSG00000004139	sterile alpha and TIR motif containing 1
SARS	ENSG00000031698	seryl-tRNA synthetase 1
SARS2	ENSG00000104835	seryl-tRNA synthetase 2 mitochondrial
SCCPDH	ENSG00000143653	saccharopine dehydrogenase (putative)
SCO1	ENSG00000133028	synthesis of cytochrome C oxidase 1
SCO2	ENSG00000284194	synthesis of cytochrome C oxidase 2
SCP2	ENSG00000116171	sterol carrier protein 2
SDHA	ENSG00000073578	succinate dehydrogenase complex flavoprotein subunit A
SDHAF1	ENSG00000205138	succinate dehydrogenase complex assembly factor 1
SDHAF2	ENSG00000167985	succinate dehydrogenase complex assembly factor 2
SDHB	ENSG00000117118	succinate dehydrogenase complex iron sulfur subunit B
SDHC	ENSG00000143252	succinate dehydrogenase complex subunit C
SDHD	ENSG00000204370	succinate dehydrogenase complex subunit D
SDR39U1	ENSG00000100445	short chain dehydrogenase/reductase family 39U member 1
SDS	ENSG00000135094	serine dehydratase
SDSL	ENSG00000139410	serine dehydratase like
SECISBP2	ENSG00000187742	SECIS binding protein 2
SELO	ENSG00000073169	selenoprotein O
SERAC1	ENSG00000122335	serine active site containing 1
SERHL2	ENSG00000183569	serine hydrolase like 2
SETD9	ENSG00000155542	SET domain containing 9
SFXN1	ENSG00000164466	sideroflexin 1
SFXN2	ENSG00000156398	sideroflexin 2
SFXN3	ENSG00000107819	sideroflexin 3
SFXN4	ENSG00000183605	sideroflexin 4
SFXN5	ENSG00000144040	sideroflexin 5
SGK1	ENSG00000118515	serum/glucocorticoid regulated kinase 1
SH3BP5	ENSG00000131370	SH3 domain binding protein 5
SHC1	ENSG00000160691	SHC adaptor protein 1
SHMT1	ENSG00000176974	serine hydroxymethyltransferase 1
SHMT2	ENSG00000182199	serine hydroxymethyltransferase 2
SIAH3	ENSG00000215475	siah E3 ubiquitin protein ligase family member 3
SIRT1	ENSG00000096717	sirtuin 1
SIRT3	ENSG00000142082	sirtuin 3
SIRT4	ENSG00000089163	sirtuin 4
SIRT5	ENSG00000124523	sirtuin 5
SIVA1	ENSG00000184990	SIVA1 apoptosis inducing factor
SLC16A1	ENSG00000155380	solute carrier family 16 member 1

SLC16A11	ENSG00000174326	solute carrier family 16 member 11
SLC16A7	ENSG00000118596	solute carrier family 16 member 7
SLC22A4	ENSG00000197208	solute carrier family 22 member 4
SLC25A1	ENSG00000100075	solute carrier family 25 member 1
SLC25A10	ENSG00000183048	solute carrier family 25 member 10
SLC25A11	ENSG00000108528	solute carrier family 25 member 11
SLC25A12	ENSG00000115840	solute carrier family 25 member 12
SLC25A13	ENSG00000004864	solute carrier family 25 member 13
SLC25A14	ENSG00000102078	solute carrier family 25 member 14
SLC25A15	ENSG00000102743	solute carrier family 25 member 15
SLC25A16	ENSG00000122912	solute carrier family 25 member 16
SLC25A17	ENSG00000100372	solute carrier family 25 member 17
SLC25A18	ENSG00000182902	solute carrier family 25 member 18
SLC25A19	ENSG00000125454	solute carrier family 25 member 19
SLC25A20	ENSG00000178537	solute carrier family 25 member 20
SLC25A21	ENSG00000183032	solute carrier family 25 member 21
SLC25A22	ENSG00000177542	solute carrier family 25 member 22
SLC25A23	ENSG00000125648	solute carrier family 25 member 23
SLC25A24	ENSG00000085491	solute carrier family 25 member 24
SLC25A25	ENSG00000148339	solute carrier family 25 member 25
SLC25A26	ENSG00000144741	solute carrier family 25 member 26
SLC25A27	ENSG00000153291	solute carrier family 25 member 27
SLC25A28	ENSG00000155287	solute carrier family 25 member 28
SLC25A29	ENSG00000197119	solute carrier family 25 member 29
SLC25A3	ENSG00000075415	solute carrier family 25 member 3
SLC25A30	ENSG00000174032	solute carrier family 25 member 30
SLC25A31	ENSG00000151475	solute carrier family 25 member 31
SLC25A32	ENSG00000164933	solute carrier family 25 member 32
SLC25A33	ENSG00000171612	solute carrier family 25 member 33
SLC25A34	ENSG00000162461	solute carrier family 25 member 34
SLC25A35	ENSG00000125434	solute carrier family 25 member 35
SLC25A36	ENSG00000114120	solute carrier family 25 member 36
SLC25A37	ENSG00000147454	solute carrier family 25 member 37
SLC25A38	ENSG00000144659	solute carrier family 25 member 38
SLC25A39	ENSG00000013306	solute carrier family 25 member 39
SLC25A4	ENSG00000151729	solute carrier family 25 member 4
SLC25A40	ENSG00000075303	solute carrier family 25 member 40
SLC25A41	ENSG00000181240	solute carrier family 25 member 41
SLC25A42	ENSG00000181035	solute carrier family 25 member 42
SLC25A43	ENSG00000077713	solute carrier family 25 member 43
SLC25A44	ENSG00000160785	solute carrier family 25 member 44
SLC25A45	ENSG00000162241	solute carrier family 25 member 45
SLC25A46	ENSG00000164209	solute carrier family 25 member 46
SLC25A47	ENSG00000140107	solute carrier family 25 member 47
SLC25A48	ENSG00000145832	solute carrier family 25 member 48
SLC25A53	ENSG00000269743	solute carrier family 25 member 53
SLC25A6	ENSG00000169100	solute carrier family 25 member 6
SLC27A2	ENSG00000140284	solute carrier family 27 member 2
SLC27A3	ENSG00000143554	solute carrier family 27 member 3
SLC30A6	ENSG00000152683	solute carrier family 30 member 6
SLC30A9	ENSG00000014824	solute carrier family 30 member 9
SLC35F6	ENSG00000213699	solute carrier family 35 member F6

SLC37A4	ENSG00000137700	solute carrier family 37 member 4
SLC8A3	ENSG00000100678	solute carrier family 8 member A3
SLC9A1	ENSG00000090020	solute carrier family 9 member A1
SLC9A6	ENSG00000198689	solute carrier family 9 member A6
SLIRP	ENSG00000119705	SRA stem-loop interacting RNA binding protein
SLIT3	ENSG00000184347	slit guidance ligand 3
SLMO1	ENSG00000141391	PRELI domain containing 3A
SLMO2	ENSG00000101166	PRELI domain containing 3B
SMAD1	ENSG00000170365	SMAD family member 1
SMIM4	ENSG00000168273	small integral membrane protein 4
SNAP29	ENSG00000099940	synaptosome associated protein 29
SNCA	ENSG00000145335	synuclein alpha
SNCB	ENSG00000074317	synuclein beta
SND1	ENSG00000197157	staphylococcal nuclease and tudor domain containing 1
SOD1	ENSG00000142168	superoxide dismutase 1
SOD2	ENSG00000112096	superoxide dismutase 2
SORD	ENSG00000140263	sorbitol dehydrogenase
SOX4	ENSG00000124766	SRY-box transcription factor 4
SPARC	ENSG00000113140	secreted protein acidic and cysteine rich
SPATA19	ENSG00000166118	spermatogenesis associated 19
SPATA20	ENSG00000006282	spermatogenesis associated 20
SPATA5	ENSG00000145375	spermatogenesis associated 5
SPG7	ENSG00000197912	SPG7 matrix AAA peptidase subunit paraplegin
SPHKAP	ENSG00000153820	SPHK1 interactor AKAP domain containing
SPR	ENSG00000116096	sepiapterin reductase
SPRYD4	ENSG00000176422	SPRY domain containing 4
SPTLC2	ENSG00000100596	serine palmitoyltransferase long chain base subunit 2
SQRDL	ENSG00000137767	sulfide quinone oxidoreductase
SRC	ENSG00000197122	SRC proto-oncogene non-receptor tyrosine kinase
SREK1	ENSG00000153914	splicing regulatory glutamic acid and lysine rich protein 1
SRP19	ENSG00000153037	signal recognition particle 19
SSBP1	ENSG00000106028	single stranded DNA binding protein 1
STAP1	ENSG00000035720	signal transducing adaptor family member 1
STAR	ENSG00000147465	steroidogenic acute regulatory protein
STARD13	ENSG00000133121	StAR related lipid transfer domain containing 13
STARD3	ENSG00000131748	StAR related lipid transfer domain containing 3
STARD7	ENSG00000084090	StAR related lipid transfer domain containing 7
STK11	ENSG00000118046	serine/threonine kinase 11
STOM	ENSG00000148175	stomatin
STOML1	ENSG00000067221	stomatin like 1
STOML2	ENSG00000165283	stomatin like 2
STX17	ENSG00000136874	syntaxin 17
STXBP1	ENSG00000136854	syntaxin binding protein 1
SUCLA2	ENSG00000136143	succinate-CoA ligase ADP-forming subunit beta
SUCLG1	ENSG00000163541	succinate-CoA ligase GDP/ADP-forming subunit alpha
SUGCT	ENSG00000175600	succinyl-CoA:glutarate-CoA transferase
SUOX	ENSG00000139531	sulfite oxidase
SUPV3L1	ENSG00000156502	Suv3 like RNA helicase
SURF1	ENSG00000148290	SURF1 cytochrome c oxidase assembly factor
SYNE2	ENSG00000054654	spectrin repeat containing nuclear envelope protein 2
SYNJ2BP	ENSG00000213463	synaptojanin 2 binding protein
TACO1	ENSG00000136463	translational activator of cytochrome c oxidase I

TANGO2	ENSG00000183597	transport and golgi organization 2 homolog
TAP1	ENSG00000168394	transporter 1 ATP binding cassette subfamily B member
TARS	ENSG00000113407	threonyl-tRNA synthetase 1
TARS2	ENSG00000143374	threonyl-tRNA synthetase 2 mitochondrial
TAT	ENSG00000198650	tyrosine aminotransferase
TATDN3	ENSG00000203705	TatD DNase domain containing 3
TAZ	ENSG00000102125	tafazzin phospholipid-lysophospholipid transacylase
TBC1D15	ENSG00000121749	TBC1 domain family member 15
TCAIM	ENSG00000179152	T cell activation inhibitor mitochondrial
TCHP	ENSG00000139437	trichoplein keratin filament binding
TCIRG1	ENSG00000110719	T cell immune regulator 1 ATPase H+ transporting V0 subunit a3
TDH	ENSG00000154316	L-threonine dehydrogenase (pseudogene)
TEFM	ENSG00000172171	transcription elongation factor mitochondrial
TFAM	ENSG00000108064	transcription factor A mitochondrial
TFB1M	ENSG00000029639	transcription factor B1 mitochondrial
TFB2M	ENSG00000162851	transcription factor B2 mitochondrial
TGM2	ENSG00000198959	transglutaminase 2
TH	ENSG00000180176	tyrosine hydroxylase
THEM4	ENSG00000159445	thioesterase superfamily member 4
THG1L	ENSG00000113272	tRNA-histidine guanylyltransferase 1 like
THNSL1	ENSG00000185875	threonine synthase like 1
TIMM10B	ENSG00000132286	translocase of inner mitochondrial membrane 10B
TIMM13	ENSG00000099800	translocase of inner mitochondrial membrane 13
TIMM17A	ENSG00000134375	translocase of inner mitochondrial membrane 17A
TIMM21	ENSG00000075336	translocase of inner mitochondrial membrane 21
TIMM22	ENSG00000177370	translocase of inner mitochondrial membrane 22
TIMM23	ENSG00000265354	translocase of inner mitochondrial membrane 23
TIMM44	ENSG00000104980	translocase of inner mitochondrial membrane 44
TIMM50	ENSG00000105197	translocase of inner mitochondrial membrane 50
TIMM8A	ENSG00000126953	translocase of inner mitochondrial membrane 8A
TIMM8B	ENSG00000150779	translocase of inner mitochondrial membrane 8 homolog B
TIMM9	ENSG00000100575	translocase of inner mitochondrial membrane 9
TIMMDC1	ENSG00000113845	translocase of inner mitochondrial membrane domain containing 1
TK2	ENSG00000166548	thymidine kinase 2
TKT	ENSG00000163931	transketolase
TMBIM4	ENSG00000155957	transmembrane BAX inhibitor motif containing 4
TMEM11	ENSG00000178307	transmembrane protein 11
TMEM126A	ENSG00000171202	transmembrane protein 126A
TMEM126B	ENSG00000171204	transmembrane protein 126B
TMEM143	ENSG00000161558	transmembrane protein 143
TMEM14C	ENSG00000111843	transmembrane protein 14C
TMEM160	ENSG00000130748	transmembrane protein 160
TMEM177	ENSG00000144120	transmembrane protein 177
TMEM186	ENSG00000184857	transmembrane protein 186
TMEM205	ENSG00000105518	transmembrane protein 205
TMEM70	ENSG00000175606	transmembrane protein 70
TMEM8B	ENSG00000137103	transmembrane protein 8B
TMLHE	ENSG00000185973	trimethyllysine hydroxylase epsilon
TMTC1	ENSG00000133687	transmembrane O-mannosyltransferase targeting cadherins 1
TNNC1	ENSG00000114854	troponin C1 slow skeletal and cardiac type
TOMM20	ENSG00000173726	translocase of outer mitochondrial membrane 20
TOMM22	ENSG00000100216	translocase of outer mitochondrial membrane 22

TOMM34	ENSG00000025772	translocase of outer mitochondrial membrane 34
TOMM40	ENSG00000130204	translocase of outer mitochondrial membrane 40
TOMM40L	ENSG00000158882	translocase of outer mitochondrial membrane 40 like
TOMM5	ENSG00000175768	translocase of outer mitochondrial membrane 5
TOMM6	ENSG00000214736	translocase of outer mitochondrial membrane 6
TOMM7	ENSG00000196683	translocase of outer mitochondrial membrane 7
TOMM70A	ENSG00000154174	translocase of outer mitochondrial membrane 70
TOP1MT	ENSG00000184428	DNA topoisomerase I mitochondrial
TOP3A	ENSG00000177302	DNA topoisomerase III alpha
TP53	ENSG00000141510	tumor protein p53
TP53AIP1	ENSG00000120471	tumor protein p53 regulated apoptosis inducing protein 1
TPI1	ENSG00000111669	triosephosphate isomerase 1
TPO	ENSG00000115705	thyroid peroxidase
TPP1	ENSG00000166340	tripeptidyl peptidase 1
TRAF3	ENSG00000131323	TNF receptor associated factor 3
TRAF6	ENSG00000175104	TNF receptor associated factor 6
TRAK1	ENSG00000182606	trafficking kinesin protein 1
TRAK2	ENSG00000115993	trafficking kinesin protein 2
TRAK2	ENSG00000115993	trafficking kinesin protein 2
TRAP1	ENSG00000126602	TNF receptor associated protein 1
TRIAP1	ENSG00000170855	TP53 regulated inhibitor of apoptosis 1
TRIM31	ENSG00000204616	tripartite motif containing 31
TRIM39	ENSG00000204599	tripartite motif containing 39
TRIT1	ENSG00000043514	tRNA isopentenyltransferase 1
TRMT10C	ENSG00000174173	tRNA methyltransferase 10C mitochondrial RNase P subunit
TRMT11	ENSG00000066651	tRNA methyltransferase 11 homolog
TRMT2B	ENSG00000188917	tRNA methyltransferase 2 homolog B
TRMT61B	ENSG00000171103	tRNA methyltransferase 61B
TRMU	ENSG00000100416	tRNA mitochondrial 2-thiouridylase
TRNT1	ENSG00000072756	tRNA nucleotidyl transferase 1
TRUB2	ENSG00000167112	TruB pseudouridine synthase family member 2
TSFM	ENSG00000123297	Ts translation elongation factor mitochondrial
TSHZ3	ENSG00000121297	teashirt zinc finger homeobox 3
TSPO	ENSG00000100300	translocator protein
TST	ENSG00000128311	thiosulfate sulfurtransferase
TSTD3	ENSG00000279170	thiosulfate sulfurtransferase like domain containing 3
TTC19	ENSG00000011295	tetratricopeptide repeat domain 19
TTC3	ENSG00000182670	tetratricopeptide repeat domain 3
TUBB3	ENSG00000258947	tubulin beta 3 class III
TUFM	ENSG00000178952	Tu translation elongation factor mitochondrial
TUSC2	ENSG00000114383	tumor suppressor 2 mitochondrial calcium regulator
TUSC3	ENSG00000104723	tumor suppressor candidate 3
TXN	ENSG00000136810	thioredoxin
TXN2	ENSG00000100348	thioredoxin 2
TXNDC12	ENSG00000117862	thioredoxin domain containing 12
TXNRD1	ENSG00000198431	thioredoxin reductase 1
TXNRD2	ENSG00000184470	thioredoxin reductase 2
TYMS	ENSG00000176890	thymidylate synthetase
TYSND1	ENSG00000156521	trypsin like peroxisomal matrix peptidase 1
UACA	ENSG00000137831	uveal autoantigen with coiled-coil domains and ankyrin repeats
UBA1	ENSG00000130985	ubiquitin like modifier activating enzyme 1
UBIAD1	ENSG00000120942	UbiA prenyltransferase domain containing 1

UCP1	ENSG00000109424	uncoupling protein 1
UCP2	ENSG00000175567	uncoupling protein 2
UCP3	ENSG00000175564	uncoupling protein 3
UMPS	ENSG00000114491	uridine monophosphate synthetase
UQCC2	ENSG00000137288	ubiquinol-cytochrome c reductase complex assembly factor 2
UQCR10	ENSG00000184076	ubiquinol-cytochrome c reductase complex III subunit X
UQCR11	ENSG00000127540	ubiquinol-cytochrome c reductase complex III subunit XI
UQCRB	ENSG00000156467	ubiquinol-cytochrome c reductase binding protein
UQCRC1	ENSG00000010256	ubiquinol-cytochrome c reductase core protein 1
UQCRC2	ENSG00000140740	ubiquinol-cytochrome c reductase core protein 2
UQCRFS1	ENSG00000169021	ubiquinol-cytochrome c reductase Rieske iron-sulfur polypeptide 1
UQCRH	ENSG00000173660	ubiquinol-cytochrome c reductase hinge protein
URI1	ENSG00000105176	URI1 prefoldin like chaperone
UROS	ENSG00000188690	uroporphyrinogen III synthase
USMG5	ENSG00000173915	ATP synthase membrane subunit k
USP30	ENSG00000135093	ubiquitin specific peptidase 30
USP48	ENSG00000090686	ubiquitin specific peptidase 48
UXS1	ENSG00000115652	UDP-glucuronate decarboxylase 1
VAMP8	ENSG00000118640	vesicle associated membrane protein 8
VARS	ENSG00000204394	valyl-tRNA synthetase 1
VARS2	ENSG00000137411	valyl-tRNA synthetase 2 mitochondrial
VASN	ENSG00000168140	vasorin
VDAC1	ENSG00000213585	voltage dependent anion channel 1
VDAC2	ENSG00000165637	voltage dependent anion channel 2
VDAC3	ENSG00000078668	voltage dependent anion channel 3
VHL	ENSG00000134086	von Hippel-Lindau tumor suppressor
VWA8	ENSG00000102763	von Willebrand factor A domain containing 8
WARS2	ENSG00000116874	tryptophanyl tRNA synthetase 2 mitochondrial
WDR81	ENSG00000167716	WD repeat domain 81
WWOX	ENSG00000186153	WW domain containing oxidoreductase
XAF1	ENSG00000132530	XIAP associated factor 1
XPNPEP3	ENSG00000196236	X-prolyl aminopeptidase 3
XRCC3	ENSG00000126215	X-ray repair cross complementing 3
XRCC6BP1	ENSG00000166896	ATP23 metallopeptidase and ATP synthase assembly factor homolog
YARS2	ENSG00000139131	tyrosyl-tRNA synthetase 2
YBEY	ENSG00000182362	ybeY metalloendoribonuclease
YKT6	ENSG00000106636	YKT6 v-SNARE homolog
YME1L1	ENSG00000136758	YME1 like 1 ATPase
YRDC	ENSG00000196449	yrdC N6-threonylcarbamoyltransferase domain containing
YWHAE	ENSG00000108953	tyrosine 3-monooxygenase/tryptophan 5-monooxygenase activation protein epsilon
YWHAZ	ENSG00000164924	tyrosine 3-monooxygenase/tryptophan 5-monooxygenase activation protein zeta
ZBED8	ENSG00000221886	zinc finger BED-type containing 8
ZDHHC8	ENSG00000099904	zinc finger DHHC-type palmitoyltransferase 8
ZFHX3	ENSG00000140836	zinc finger homeobox 3
ZMIZ2	ENSG00000122515	zinc finger MIZ-type containing 2
ZNF428	ENSG00000131116	zinc finger protein 428
