# Supplementary figures and images for: Chagas Disease Megaesophagus Patients Carrying Variant MRPS18B P260A Display Nitro-Oxidative Stress and Mitochondrial Dysfunction in Response to IFN-γ Stimulus
Source: Biomedicines. 2022 Sep 7;10(9):2215. doi: 10.3390/biomedicines10092215 (PMC9496350; doi:10.3390/biomedicines10092215)

Supplemental Figure S1 : Sanger sequencing chromatograms on CME patients.

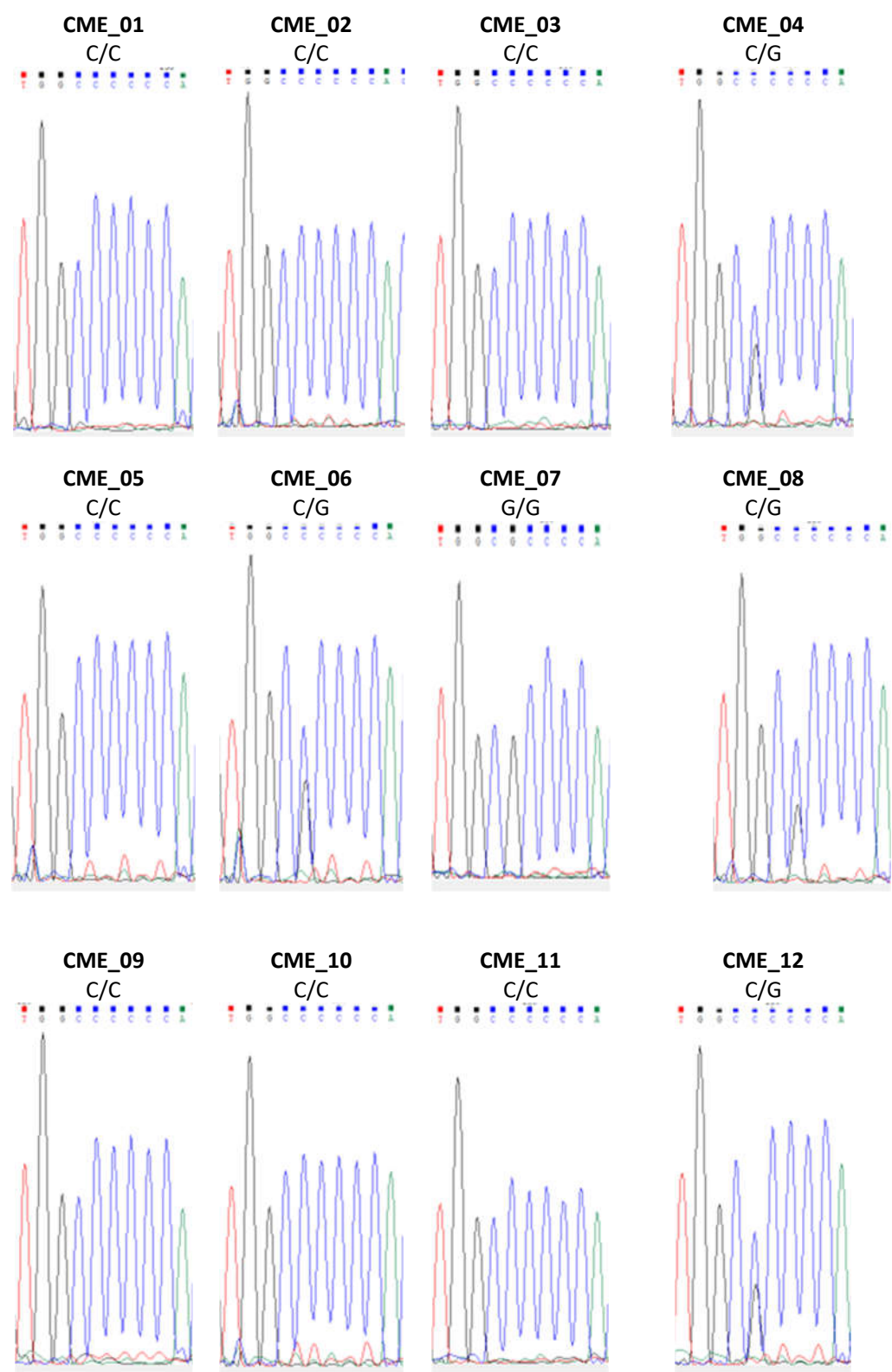

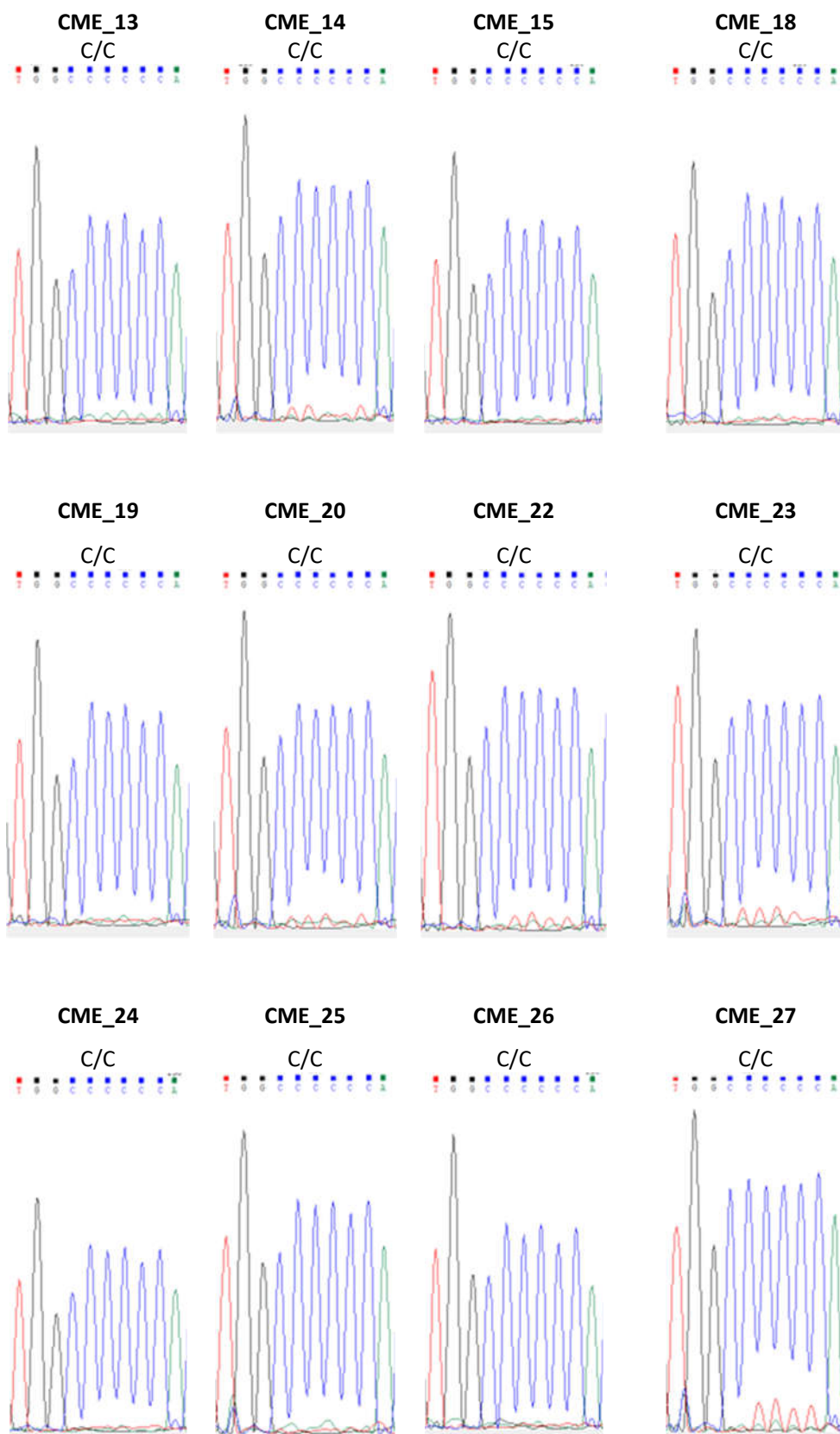

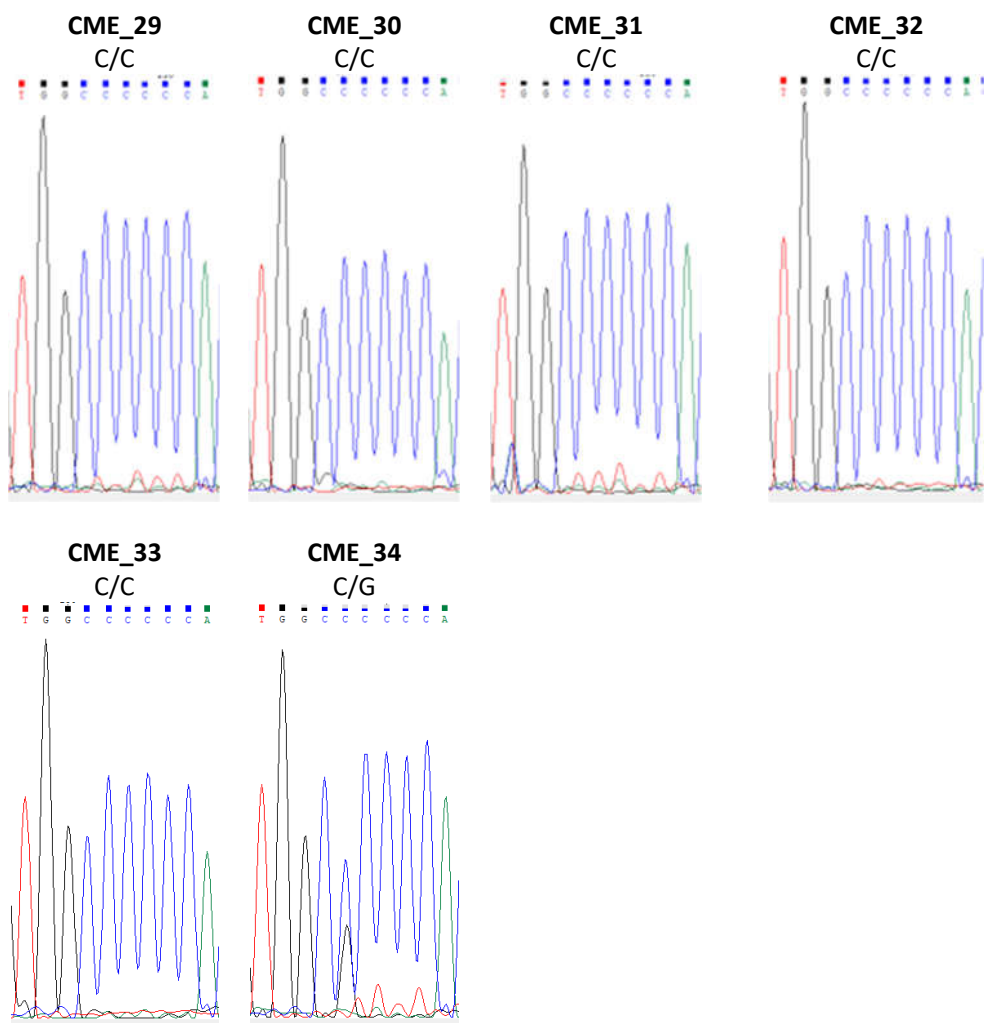

Supplement: Supplementary file 1 [file biomedicines-10-02215-s001.zip › Supplemental Figure S1.pdf]
